# DFT Investigation into the Role of Superbases as the Auxiliary Groups in CO_2_ Reduction †

**DOI:** 10.3390/molecules31071167

**Published:** 2026-04-01

**Authors:** Zoran Glasovac, Borislav Kovačević, Davor Margetić

**Affiliations:** 1 Division of Organic Chemistry and Biochemistry, Ruđer Bošković Institute, HR-10000 Zagreb, Croatia; davor.margetic@irb.hr; 2 Division of Physical Chemistry, Ruđer Bošković Institute, HR-10000 Zagreb, Croatia; borislav.kovacevic@irb.hr

**Keywords:** hydricity, DFT calculations, superbases, hydrogen bonding, CO_2_ reduction, aromaticity

## Abstract

Non-metallic hydride donors have emerged as an interesting, highly tunable class of compounds capable of CO_2_ reduction, with benzimidazoles being simple, yet efficient and regenerable, representatives. In this work, the role of superbases as auxiliary groups attached to the benzimidazole framework was investigated using the CPCM(CH_3_CN)/ωB97xD/aug-cc-pVTZ//CPCM(CH_3_CN)/ωB97xD/6-31+G(d,p) approach. Three modes of operation were assessed through hydricity calculations and the modeling of two different CO_2_ reduction mechanisms. Among the superbases considered, phosphazene substituents yielded the largest increase in the hydride donation ability, lowering hydricity by 6 kcal mol^−1^ relative to 2-methylbenzimidazole, with the α-substitution exerting a stronger effect than β-substitution. For most systems, changes in hydricity correlate with changes in aromaticity, except in systems where steric congestion limits optimal substituent alignment. CO_2_ activation pathways encompassing guanidine/CO_2_ hydrogen bonding and guanidinium carboxamidine formation were modeled. In the former, transition state structures were significantly stabilized, and the overall exergonicity of the reduction is enhanced. Also, utilizing the longer and more flexible linker additionally decreases the barrier for the reaction. The carboxamidine pathway is disfavored because of the high stability of the carboxamidine intermediate and low barrier for the C–N bond cleavage, which reverses the mechanism to the reduction of isolated CO_2_.

## 1. Introduction

Global warming is one of the greatest ecological challenges. Carbon dioxide is recognized as one of the main contributors to this phenomenon as its atmospheric concentration continuously increases [1]. Much effort has been devoted to optimizing the circular economy of CO_2_, thereby reducing the carbon footprint as much as possible. In general, the conversion of CO_2_ into value-added chemicals represents an important scientific challenge, and researchers across all fields are working hard to achieve this goal while preserving process sustainability and adhering to the 12 principles of green chemistry [2]. Among various strategies, treating CO_2_ as a feedstock chemical for polymer processing and liquid fuel production is considered a highly promising use [3].

Despite its high thermodynamic stability and significant electrochemical potential needed for its reduction, CO_2_ can be converted into valuable organic chemicals both electrochemically and non-electrochemically using appropriate activators [4,5,6,7]. The non-electrochemical strategies commonly used today involve the reaction of CO_2_ with strong nucleophiles, such as hydride anions, metal hydrides, and superbases. Studies indicate that amines and other nitrogen-based nucleophiles form carbamate intermediates; these are essential in the synthesis of organic carbonates and polycarbonates, which serve as green solvents or biodegradable polymers [8,9,10,11,12,13]. In this context, guanidines—as archetypal superbases—exhibit high catalytic activity while remaining cost-effective, readily accessible, and free of heavy metal ions [10,14]. Besides that, cyclic guanidines have been recognized as effective catalysts for the CO_2_ reduction with hydrides [15,16], as well as potent hydride donors [17,18]. It has been proposed that the guanidinium cation effectively stabilizes the development of the negative charge during CO_2_ reduction [19]. Motivated by this idea, a guanidine-rich metal–organic cage (MOC) was designed and employed as the active catalyst for the electrochemical activation of CO_2_ [20].

Bioinspired organic hydrides have emerged as a promising class of reductants capable of converting CO_2_ into formic acid and its derivatives. These species are particularly attractive because they can be regenerated through electrochemical reduction [21]. Their capacity to transfer hydride anion to an acceptor is determined by hydricity (ΔG°_H^−^_), defined as the Gibbs energy for the hydride transfer half-reaction (Equation (1)).

DH → D^+^ + H^−^  Δ_r_*G*° = Δ*G*°_H^−^_(DH) (1)

**Scheme 1:**
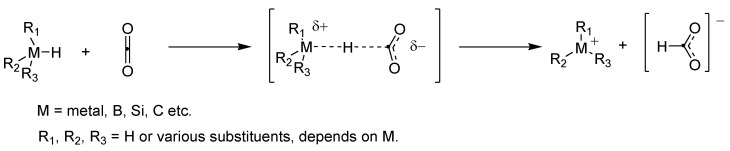
General reaction scheme for the reduction of CO_2_ with the neutral hydride donor.

The experimental hydricity of CO_2_ in acetonitrile was determined to be 184 kJ mol^−1^ (44 kcal mol^−1^), and it was measured by bracketing against [HPt(diphosphine)_2_]^+^ hydride donors [22,23]. The hydride donors (DH) with hydricity values lower than this threshold are thermodynamically capable of reducing CO_2_ to formic acid. The general mechanism for this process employing a neutral hydride donor is illustrated in Scheme 1. As noted by Glusac and coworkers, mechanisms such as aromatization or the addition of electron-donating substituents stabilize the resulting cation and shift the equilibrium toward the products [21].

Guanidines and other strong bases are recognized as powerful π–electron donors and employed as the building blocks in the design of highly basic phosphines [24,25]. Analogous to the effect of NMe_2_ on the hydricity of the benzimidazole derivatives [26], and the recent results in the design of bisphosphine-type hydride donors [27], the incorporation of superbasic groups into the hydride donor framework is expected to lower their hydricity (**Role 1**). Furthermore, given the beneficial role of the guanidinium cation in stabilizing charge, we envisioned that it could serve as a functional auxiliary group. Specifically, we postulate that it may promote hydride transfer to CO_2_ by stabilizing the transition state and the final product through hydrogen bonding with CO_2_ and formate anion (**Role 2**), thereby increasing the reaction rate and overall exergonicity of the reaction. Alternatively, neutral guanidines are known to bind CO_2_ and form carboxamidines (**Role 3**). This process eliminates the energetic penalty for CO_2_ deformation during reduction and may lower the kinetic barrier [15].

This study explores how guanidine and related superbases affect the hydricity of benzimidazole derivatives, focusing specifically on aromaticity as a primary driver of cation stabilization and its changes upon the hydride transfer. Additionally, we evaluate the viability of guanidines in cooperative CO_2_ reduction through a detailed analysis of the most probable reaction pathways.

## 2. Results

### 2.1. Influence of Superbases on the Hydricity of the Selected Benzimidazole Derivatives (Role 1)

**Figure 1:**
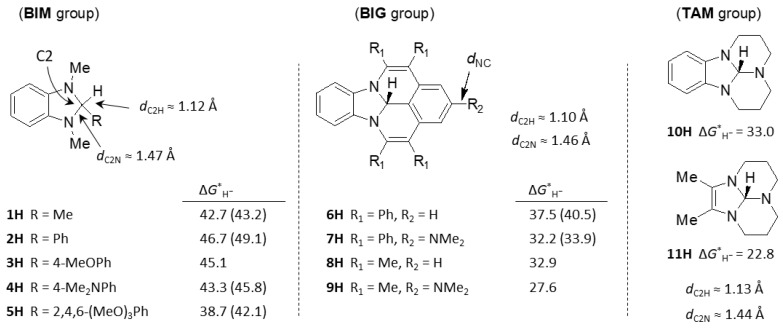
Structures, hydricities, and the selected geometrical parameters of the three groups of benzimidazole derivatives calculated using the CPCM(CH_3_CN)/ωB97xD/aug-cc-pVTZ//CPCM(CH_3_CN)/ωB97xD/6-31+G(d,p) approach. Hydricities (ΔG*_H^−^_) are given in kcal mol^−1^ (1 kcal = 4.184 kJ); experimental values (in DMSO) are given in parentheses.

The analysis started by comparing the hydricities of known hydride donors based on the benzimidazole framework (**1H**–**7H**). The initial set of benzimidazole systems (**BIM** group) was further enlarged to include hexacyclic analogs **8H** and **9H** (**BIG** group) and triaminomethane derivatives **10H** and **11H** (**TAM** group). This selection covers three distinct cation stabilization modes: (i) varying the π-electron donation power at position 2 of the imidazole subunit (**1H**–**5H**), (ii) extended delocalization over the polycyclic backbone (**6H**–**9H**), and (iii) formation of a tricyclic guanidium cation (**10H** and **11H**). The hydricities for all compounds were calculated in acetonitrile at the CPCM(CH_3_CN)/ωB97xD/aug-cc-pVTZ//CPCM(CH_3_CN)/ωB97xD/6-31+G(d,p) level of theory. This computational approach demonstrated very good correlation between the experimental and computed hydricities [17,21,28]. The structures and the calculated data are presented in Figure 1, while the energy data are given in Table S1a (Supplementary Materials).

The calculated hydricities for **1H**, **2H,** and **4H**–**7H** are, on average, 3 kcal mol^−1^ lower than the experimental data measured in DMSO [26], while the relative trend is well preserved with a correlation coefficient of *r*^2^ = 0.967 (Figure S1). Replacing the phenyl subunit with 4-NMe_2_Ph (going from **2H**, **6H**, and **8H** to **4H**, **7H**, and **9H**) decreases the hydricity by 3.5, 5.6, and 5.6 kcal mol^−1^, respectively. The more pronounced substituent effect observed in **7H**/**7^+^** and **9H**/**9^+^** systems is expected, as these cations adopt a fully planar conformation. Planarity facilitates efficient charge delocalization across the entire carbocyclic framework and maximizes the π-donating contribution of the NMe_2_ group. In contrast, the phenyl ring in **2^+^** and **4^+^** is nearly perpendicular to the benzimidazole plane, which restricts charge transfer. This is qualitatively evidenced by the shortening of the *d*_NC_ bond (Figure 1) by 0.028 Å in **7H** to **7^+^** or **9H** to **9^+^**, compared to 0.020 Å for the **4H**/**4^+^** pair. Additionally, NBO analysis indicates that nitrogen lone-pair donation in **4^+^** (quantified by the *E*^2^_ij_ stabilization energy in the NBO framework) is 3–4 kcal mol^−1^ smaller than in the other two cations. This finding supports a more substantial increase in the double-bond character of the *d*_NC_ bond in the fully planar systems.

**Figure 2:**
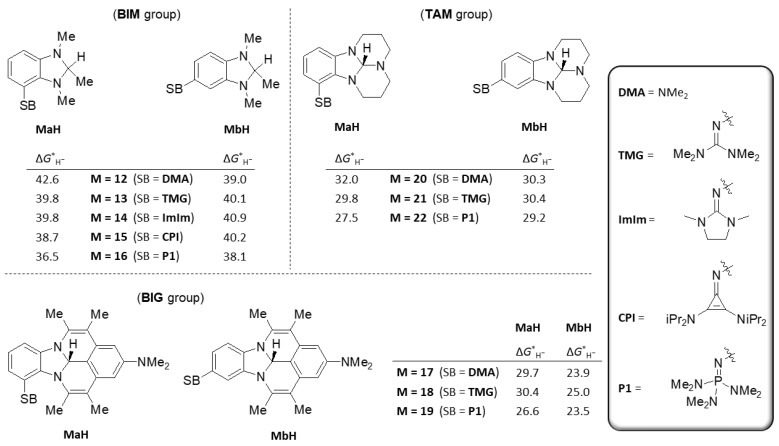
Structures and hydricities of α- and β-substituted benzimidazole derivatives calculated using the CPCM(CH_3_CN)/ωB97xD/aug-cc-pVTZ//CPCM(CH_3_CN)/ωB97xD/6-31+G(d,p) approach. Hydricities (ΔG*_H^−^_) are given in kcal mol^−1^ (1 kcal = 4.184 kJ).

Three groups of hydride donors (**BIM**, **BIG**, and **TAM**) were further expanded to include derivatives bearing dimethylamino (**DMA**) or superbase substituents at the α- and β-position of the benzimidazole subunit. Electron-rich superbases were chosen because they are expected to lower hydricity by stabilizing the cation formed after the hydride transfer. The structures are shown in Figure 2, and energies are given in Table S1b (Supplementary Materials).

Incorporating dimethylamino or superbasic groups into the benzimidazole subunit generally decreases hydricity (Figure 1 and Figure 2), with a more pronounced effect observed for derivatives (**MaH**) compared to β-analogs (**MbH**). Among the groups studied, the influence of the **P1** phosphazene substituent was the largest. For instance, substitution with the **P1** group at the α- and β-positions lowers the hydricity of **1H** by 6.2 and 4.6 kcal mol^−1^, respectively. While the two guanidine moieties (**TMG** and **ImIm**) show similar impacts on ΔG*_H^−^_(**1H**), the effect of **CPI** falls between that of **P1** and **TMG**. Interestingly, the **DMA** group exhibits the opposite trend: hydricities for α-substituted derivatives (**12aH**, **17aH**, and **20aH**) are equal to or higher than those calculated for the parent structures. Conversely, **DMA** substitution at the β-position lowers the hydricities by up to 3.7 kcal mol^−1^; the effect is slightly weaker than **P1** but larger than that of **TMG**, **ImIm**, or **CPI**.

**Figure 3:**
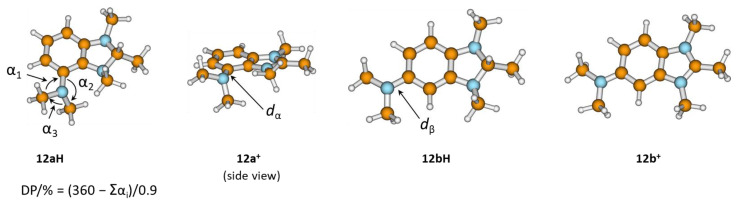
Structures of **12aH**, **12bH**, and their cations optimized using the CPCM(CH_3_CN)/ωB97xD/aug-cc-pVTZ//CPCM(CH_3_CN)/ωB97xD/6-31+G(d,p) approach with emphasis on the pyramidalization of the α–DMA group.

The reduced effectiveness of α-substitution stems from the inability of the **DMA** group to remain coplanar with the aromatic ring. Consequently, a nitrogen lone-pair is rotated to 49° (42° for the cation) relative to the benzene ring plane. Also, the degree of pyramidalization (**DP**) at the nitrogen atom of the **DMA** group is significant, measuring 20.1° (**12aH**) and 21.0° (**12a^+^**) (Figure 3). These geometrical constraints diminish π-conjugation, echoing patterns observed in strained guanidines [29]. Inspection of the geometries of **16aH** and **16bH** reveals that the optimal conformation favors π–π rather than n–π overlap across the substituent–framework (C-SB) junction bond. This is supported by the frontier orbital analysis of the parent structures **P1H**, **TMGH,** and **CPIH,** where HOMO is π-orbital delocalized across the guanidine or phosphazene double bond (Supplementary Materials, Figure S2). Due to the quasi-spherical geometry of the **P1** group, structures **16aH** and **16bH**, and their cationic forms, can achieve a coplanar arrangement of the π- systems, facilitating the effective delocalization across the junction bond. Furthermore, the nitrogen lone-pair in **16a** is oriented toward the N_im_-Me methyl group, forming an [N∙∙∙H-C] hydrogen bond. This interaction is supported by the identification of corresponding (3,−1) bond critical points (BCPs) along both its covalent and noncovalent bond paths (Supplementary Materials, Figure S3). In contrast, the geometry of the **TMG**, **ImIm,** and **CPI** groups hinders planarization and enforces the less effective n–π delocalization. A similar rationalization applies to the β-substituted derivatives. In these systems, the **DMA** and **P1** groups can adopt geometries that maximize π-donation, resulting in lower hydricities than those of the **TMG**, **ImIm**, and **CPI** derivatives. Despite the significant changes in hydricities caused by the substitution, no significant changes in the partial charges and the C2-H bond lengths in either the **BIM** or **TAM** groups of compounds were observed (Table S2 in Supplementary Materials).

The hydricity differences between **22aH** and **22bH** are essentially identical to those observed for **16aH** and **16bH**, suggesting that similar geometric and electronic effects are at play. Conversely, the **BIG** family of structures exhibits an opposing trend. In these systems, significant steric congestion arises from the proximity of the lateral backbone methyl groups to the superbasic subunit. Consequently, the hydricities of the **17bH**–**19bH** derivatives are lower than those of their corresponding α-isomers.

For the next step, the impact of the substitution on aromaticity was examined, utilizing the MCI [30] and NICS(1)_zz_ [31] indices. While no general correlation between these indices was observed, the results are consistent with known trends. Krygowski and coworkers previously established that strongly aromatic systems are generally resistant to the substituent effect, becoming more sensitive as the aromaticity decreases [32]. Consequently, the observed variation within 7 units for NICS and 1.5 units for MCI is not surprising. Both indices confirm that all studied systems remain highly aromatic, with a slight increase in the aromaticity of the benzene ring following hydride transfer; additionally, the imidazolidine ring gains aromatic character as expected. Substituents attached to the benzimidazole subunit generally reduce aromaticity, with **P1** exerting the most significant influence. The changes in the indices are more pronounced for the cationic structures than for neutrals, a finding that seems to oppose the claims of Krygowski and coworkers mentioned above, but this phenomenon needs to be re-examined in more detail on a larger set of molecules. Finally, it is noteworthy that β-substitution triggers more substantial changes in aromaticity than α-substitution (Figure S4 in Supplementary Materials).

Having confirmed that certain structural groups exhibit systematic changes in aromaticity upon substitution, we investigated whether this trend consistently extends to hydricity. For this purpose, we calculated total changes in aromaticity (Δ(MCI), Equations (2)–(4)) and compared the values with the calculated ΔG*_H^−^_ (Figure 1 and Figure 2).

Δ(MCI)_6_ = MCI(DH)_6_ − MCI(D^+^)_6_ (2)

Δ(MCI)_5_ = MCI(DH)_5_ − MCI(D^+^)_5_ (3)

**Figure 4:**
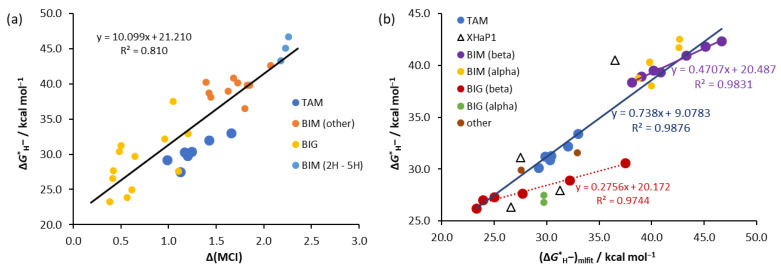
Correlation of the directly calculated ΔG*_H^−^_ of investigated hydride donors against (**a**) total change in the MCI indices (Equation (4)), and (**b**) ΔG*_H^−^_ values obtained by multiple linear regression using Δ(MCI)_6_ and Δ(MCI)_5_ indices as the independent variables (Equation (5)).

Δ(MCI) = Δ(MCI)_6_ + Δ(MCI)_5_ (4)

(ΔG*_H^−^_)_fit_ = 5.01 × Δ(MCI)_6_ + 9.96 × Δ(MCI)_5_ + 18.45 (5)

where indices 5 and 6 refer to the 5- and 6-membered rings, respectively. Equation (5) was derived via multiple linear regression using two independent variables (Δ(MCI)_6_ and Δ(MCI)_5_) across the set of 35 investigated hydride donors. The resulting data are tabulated in Tables S3 and S4 (Supplementary Materials), and the observed trends are shown in Figure 4.

While both approaches show systematic changes for specific groups of compounds, multiple linear regression indicates better overall agreement. The parent structures and their β-substituted derivatives generally correlate well with the established trend line. In contrast, α-substitution results in significant deviation from the correlation line, except for most members of the **TAM** series. However, α-**P1**-substituted derivatives (outlined triangles in Figure 4) consistently act as outliers in all categories, including **TAM**. Significant outliers and a distinct regression slope are observed for structures where the cation is delocalized over large polycyclic systems (**BIG** group, red dots in Figure 4). This divergence suggests that their electronic properties are governed by factors requiring further investigation. These trends demonstrate that the perturbations in aromaticity induced by the substitution systematically influence hydricity.

### 2.2. CO_2_ Reduction Assisted by Guanidinium Cation (Role 2)

**Figure 5:**
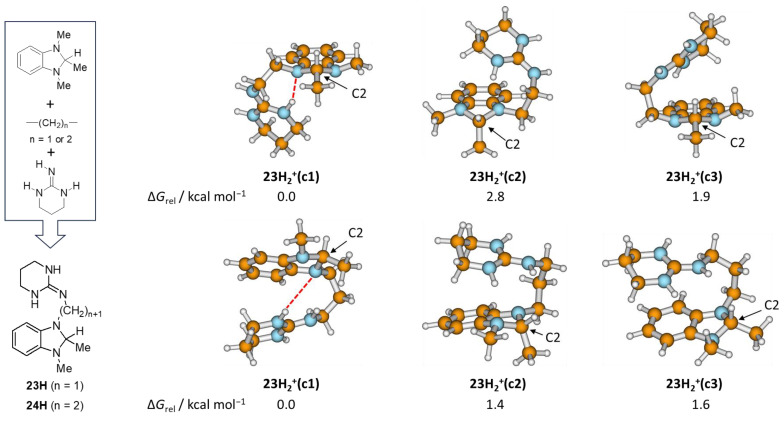
Structures and the relative stabilities of three conformers of **23H_2_^+^** and **24H_2_^+^** optimized using the CPCM(CH_3_CN)/ωB97xD/aug-cc-pVTZ//CPCM(CH_3_CN)/ωB97xD/6-31+G(d,p) approach.

The second role of the superbases examined in this work is their potential application as activators of CO_2_ reduction through hydrogen bonding interactions. Our selection was restricted to a protonated guanidine capable of forming two hydrogen bonds with the formate anion. The influence of the guanidinium cation on the kinetics and thermodynamics of CO_2_ reduction was investigated using **1H** as the hydride-donating component tethered to a tetrahydropyrimidin-2(1H)-imine via ethylene or propylene bridges, as shown in Figure 5.

A preliminary conformational search was performed using the xTB/GOAT approach, [33,34] implemented in the ORCA 6.1 software [35,36]. Twelve lowest-energy conformations were re-optimized at the ωB97xD/6-31+G(d,p) level of theory in combination with the CPCM(ACN) continuum solvation model (Section S4, Table S5a–c in Supplementary Materials). The resulting conformations for the neutral (**23H** or **24H**) and protonated (**23H_2_^+^** and **24H_2_^+^**) forms were divided into three distinct sets based on the pyramidalization of the imidazole nitrogen atoms and the position of the guanidine subunit relative to the C2-H bond. The search for the lowest-energy structures was further performed by varying the spacer conformation (gauche vs. all-antiperiplanar), the tautomeric form (endocyclic vs. exocyclic C=N double bonds in the neutral guanidine subunit), and the syn/anti isomerism at the exocyclic nitrogen atom.

Regardless of the spacer length, the most stable structures (c1 conformer, Figure 5) feature the guanidinium cation preferentially positioned “below” the benzimidazole ring (C2-Me side). The structures are characterized by the intermolecular hydrogen bonding between the guanidine and benzimidazole moieties, as evidenced by NBO second-order perturbation energies (*E*_ij_^(2)^ = 14.42 kcal mol^−1^). Additionally, conformations c2 and c3 (Figure 5) were evaluated to account for the expected synergistic effect of the two subunits during the CO_2_ binding and reduction. Conversely, in the lowest-energy conformations of **23H** and **24H**, the neutral guanidine subunit resides “above” the plane (C2-H side). Notably, these most stable neutral conformers closely correspond to conformer c3 of the protonated forms.

In the context of CO_2_ reduction, the guanidinium subunit in conformer c1 cannot assist the CO_2_ binding but influences reaction thermodynamics by modulating the hydricity of the benzimidazole fragment. Specifically, calculated hydricities of **23H** and **24H** are comparable to that of **1H**, amounting to 41.1 and 43.8 kcal mol^−1^, respectively, implying their similar capacity to reduce CO_2_. On the other hand, hydricities of their protonated forms are 51 and 47 kcal mol^−1^, respectively (Table S6 in Supplementary Materials). This shift can be attributed to the energetically unfavorable formation of the dictations.

**Figure 6:**
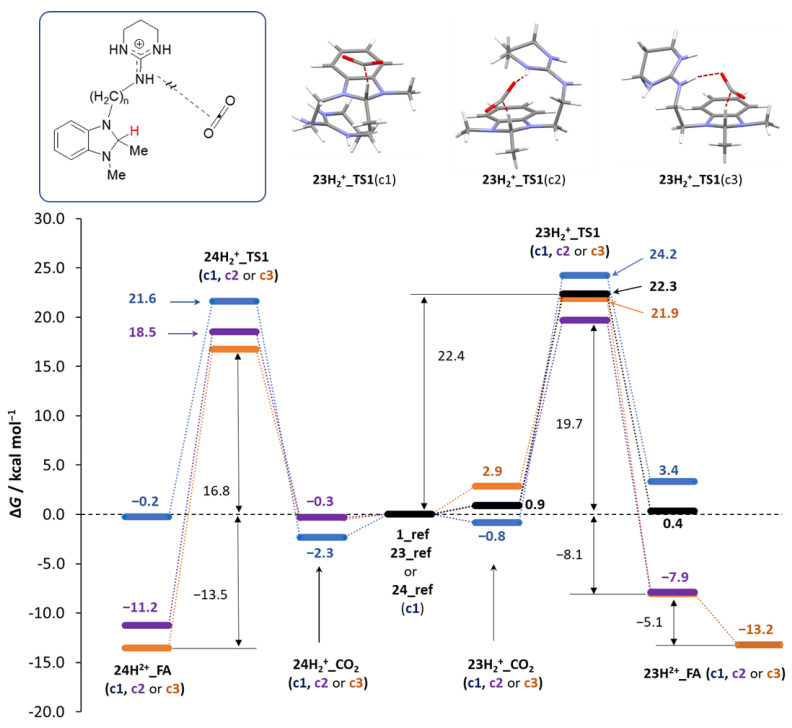
Reaction profiles for the CO_2_ reduction starting from three different conformers of **23H_2_^+^** and **24H_2_^+^** obtained from CPCM(CH_3_CN)/ωB97xD/aug-cc-pVTZ//CPCM(CH_3_CN)/ωB97xD/6-31+G(d,p) calculations. TS structures **23H_2_^+^_TS1**(c1–c3) are shown atop the profile, while the structures of **24H_2_^+^_TS1**(c1–c3) are presented in Figure S6 in the Supplementary Materials.

The CO_2_ reduction mechanism was modeled by following the reaction coordinate for hydride transfer from the benzimidazole subunit to CO_2_. The resulting barriers and reaction Gibbs energies were evaluated against the parent **1H**. Given that c2 and c3 are interconvertible via imidazole ring puckering, they likely share a transition state for the reduction step. All energies were calculated relative to the lowest-energy conformer with CO_2_ separated by 10 Å (**23_ref** and **24_ref**), the distance at which no intermolecular interactions are expected. The reaction schemes and thermodynamic profiles are summarized in Figure 6, while the energies of all species are presented in Tables S7 and S8 in the Supplementary Materials.

The lowest transition state (TS) energies were obtained for conformers c2 (**23H_2_^+^_TS**) and c3 (**24H_2_^+^_TS**), amounting to 19.8 and 16.8 kcal mol^−1^, respectively. These values are noticeably lower than in **1H_TS**. In contrast, the energy of the **23H_2_^+^_TS(c1)** conformer is 1.9 kcal mol^−1^ higher than that of **1H_TS**, while the calculated barrier for **24H_2_^+^** is nearly the same as that of **1H_TS**. Here, we recall that the guanidinium cation—CO_2_ hydrogen bond in c1 conformers is absent. These results indicate that hydrogen bonding and greater conformational flexibility are beneficial for the reduction process. We should emphasize that our calculated Δ*G*^‡^(**1H_TS**) is slightly higher than the previously reported value (Δ*G*^‡^(**1H_TS**) = 20.3 kcal mol^−1^) [37]. A discrepancy arises from the treatment of the entropy contribution, which can be a significant source of error [38]. However, as this study focuses on relative trends, any potential systematic errors are expected to be consistent across all structures. Here, we can mention that the sum of the Gibbs energies of **1H** and **CO_2_** is 13.2 kcal mol^−1^ lower than our reference point, which would lead to a much higher barrier (28.8 kcal mol^−1^) for CO_2_ reduction, comparable to that obtained by Lee and coworkers [17].

Beyond their superior kinetics, the product formed from c2 or c3 conformers of **23H_2_^+^** is more stable than that from the c1 isomer by more than 11 kcal mol^−1^. Furthermore, the spatial orientation of the guanidinium group in the c3 conformer (Figure 5) facilitates the reorientation of the resulting formate, allowing for the formation of two hydrogen bonds. This interaction additionally stabilizes the product by 5 kcal mol^−1^, resulting in an overall exergonicity of −13.2 kcal mol^−1^. Similarly, the reduction of CO_2_ with **24H_2_^+^**(c3) is exergonic by −13.5 kcal mol^−1^. However, in this case, two hydrogen bonds were formed during the IRC calculations, with no evidence of another minimum.

### 2.3. CO_2_ Reduction via Carbamates (Role 3)

**Scheme 2:**
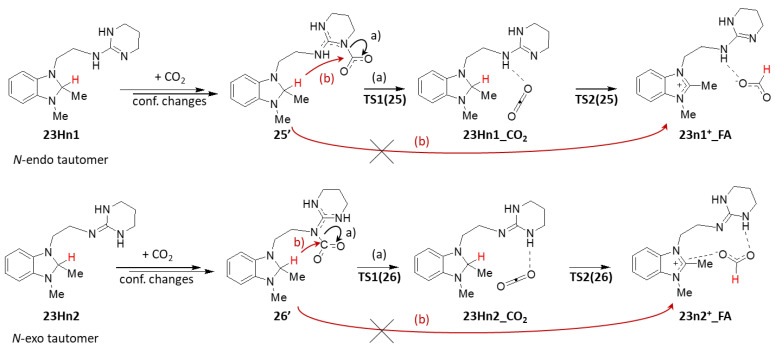
The proposed CO_2_ reduction mechanisms via the carboxamidine intermediate. (a) “two–step” mechanism with the cleavage of the carboxamidine; (b) “direct” mechanism—reduction at the carboxamidine stage.

**Figure 7:**
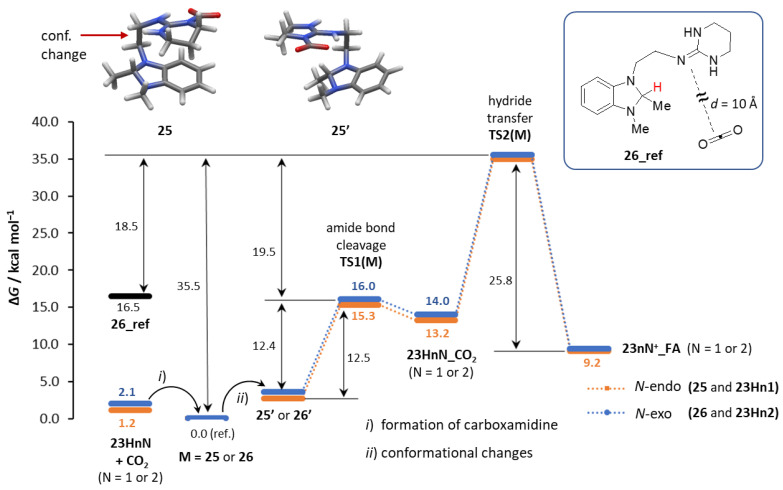
Reaction profiles for CO_2_ reduction via carbamate formation obtained from CPCM(CH_3_CN)/ωB97xD/aug-cc-pVTZ//CPCM(CH_3_CN)/ωB97xD/6-31+G(d,p) calculations. Structure **26_ref** contains CO_2_ separated from the rest of the molecule by 10 Å.

The reaction profile for CO_2_ reduction via intramolecular hydride transfer is illustrated in Scheme 2 and Figure 7. As in the previous section, the energies of all minima were referenced to the CO_2_-separated structure (**26_ref**, Figure 7 and Table S8 in Supplementary Materials). The calculations show that the most stable conformers of carboxamidines **25** and **26**, formed from two tautomeric forms of **23H**, are essentially isoenergetic and lie 16.5 kcal mol^−1^ below the CO_2_-separated structure (**26_ref**). Accordingly, the energies of the intermediates and the products, as well as activation barriers, were evaluated relative to **25** and **26**. The results indicate that the reaction does not take place at the carboxamidine stage. Instead, attempts to locate a transition state for hydride attack at the carboxamidine carbon led to C–N bond cleavage and identification of the **TS2(M)** structure. IRC calculations starting from **TS2(M)** converged to the expected products and to the intermediate complexes **23Hn1_CO_2_** or **23Hn2_CO_2_** (Figure 7), which can subsequently undergo intramolecular hydride transfer through transition states located 21 kcal mol^−1^ above these intermediates. However, due to the stability of the carboxamidines **25** and **26**, the calculated barriers for the reduction step are 35.5 kcal mol^−1^ for **TS2(25)** and 35.0 kcal mol^−1^ for **TS2(26)**, which are 11–15 kcal mol^−1^ higher than those obtained for **23H_2_^+^** (Figure 6). To complete the reaction profile, the **TS1(25)** and **TS1(26)** structures were also optimized. The calculated barriers for the formation of **25** and **26** from the intermediate complexes **23Hn1_CO_2_** and **23Hn2_CO_2_** are approximately 2 kcal mol^−1^, whereas the C-N bond cleavage (the reverse process) is significantly more demanding, with calculated barriers of 11.6 and 13.9 kcal mol^−1^, respectively.

These results indicate that, if reduction occurs, it proceeds via the noncovalent **23H_CO_2_** complex (path “a” in Scheme 2) rather than through the carboxamidine intermediate (path “b” in Scheme 2). Overall, within the proposed mechanism, the presence of neutral guanidine leads to a bifurcation of the CO_2_ pathway, with carboxamidine formation emerging as the dominant process. For the conversion of CO_2_ to formate to take place, a tandem sequence involving carboxamidine cleavage followed by hydride transfer would be required. However, the thermodynamics of this sequence are highly unfavorable, being endergonic by 13–14 kcal mol^−1^ (the initial step) and 9 kcal mol^−1^ (the overall process). Given both the relatively high activation barrier and the endergonic nature of the overall transformation, CO_2_ reduction would require significantly harsher reaction conditions. Taken together, these computational findings suggest that carboxamidine formation inhibits rather than promotes CO_2_ reduction, and that neutral guanidines are not suitable candidates for the auxiliary groups.

## 3. Discussion

This study explores the utility of guanidine and other superbases as auxiliary functional groups that can assist in the reduction of CO_2_ using non-metallic, benzimidazole-based hydride donors. Two different motifs of substitution were considered: (i) direct substitution on the benzimidazole subunit, and (ii) a guanidine substituent linked to the benzimidazole moiety by a linker.

The first type of substitution primarily modulates the hydricity, which was taken as an indication of the substituent effect. Based on the “push–pull” concept developed by Raczynska and Gal [39], it was hypothesized that a stronger π-donating group would lead to a greater decrease in hydricity. This reasoning is further supported by recent results on bisphosphines, in which P1-substituted phosphines exerted a greater effect on hydricities than TMG and amidine groups [27]. In our case, the following trend in substituent effects was observed for the β-substitution pattern: **P1** > **DMA** > **TMG** ≈ **ImIm** ≈ **CPI**. This trend is unexpected, given the trend in the basicity of phosphines and phosphazenes with the same electron-donating substituents, in which the **DMA** substituent has the weakest influence [25,40,41]. According to the results presented in the previous section, this is most likely due to the inability of the guanidine-based and CPI substituents to achieve the conformation with the optimal π-π overlap across the C-N junction bond. A more detailed insight into this observation requires a rigorous analysis of the electronic structure in these systems and in properly selected analogs, which is beyond the scope of this paper. In contrast, α-substitution yielded less predictable trends due to steric congestion, which prevents the substituents from adopting the optimal geometry for π-donation. Nevertheless, the α-substitution exerted a more pronounced impact on hydricity, with α–**P1** (**16aH**) showing a maximal decrease of 6.2 kcal mol^−1^ relative to **1H**. Interestingly, the changes in partial charges at C2 and the adjacent hydrogen atoms remain practically constant. The attenuated effects were observed in systems in which the resulting cation could delocalize over a larger aromatic framework, prompting an analysis of changes in aromaticity. While specific groups of molecules showed reasonably strong correlations, the systematic deviations observed in α–P1 derivatives suggest that a more detailed dissection of energy changes is required.

The second substitution motif introduces monocyclic guanidine or its protonated form as an auxiliary group that can assist in CO_2_ activation during the reduction step. In agreement with data from the literature [37], transition state structures for CO_2_ reduction exhibit pronounced bending of the O=C=O angle (145–148°), indicative of significant substrate activation. However, a substantial decrease in the activation barrier by 5.5 kcal mol^−1^ is observed only when a propylene linker is employed. Assuming the same pre-exponential factor, the rate constant would approximately increase by a factor of 10^4^. In contrast, the shorter ethylene linker appears insufficient to provide the conformational flexibility required to achieve optimal geometry during the reduction step.

The results also indicate that hydrogen-bonding interactions exert a stronger influence on the reaction’s thermodynamics than on its kinetics. Formation of the HB with the formate anion lowers the Gibbs energy of the reaction by up to 11 kcal mol^−1^ if two HBs are formed. Our computations further suggest that formation of the carboxamidine intermediate may serve as a side pathway that effectively sequesters CO_2_ and maintains its proximity to the reaction center. The carboxamidine moiety is cleaved during the reduction step, and the overall process proceeds similarly to the noncovalent complexes described above (Role 2). However, activation barriers are higher and have pronounced endergonicity. These findings strongly suggest that the protonated guanidine is a more effective auxiliary group than its neutral counterpart, thereby limiting the structural variability achievable through combining the different functional roles of the superbasic groups investigated in this work. Notably, the basicity of the guanidine subunit limits its possible combination with substituents playing Role 1 to those of lower basicity than guanidine itself.

## 4. Materials and Methods

*Computational details*: All DFT calculations were performed using the Gaussian16 software [42], while the conformational analysis was done using the GOAT routine implemented in the ORCA 6.1 program package [33,34,35,36]. The structures were fully optimized using ωB97xD density functional [43] in combination with the 6-31+G(d,p) basis set. The nature of the stationary points was verified with vibrational analysis (NImag = 0 for minima or NImag = 1 for the TS structures). Electronic energies were refined by the single-point calculations at the ωB97xD/aug-cc-pVTZ levels of theory. Calculations were performed in acetonitrile approximated as the dielectric continuum, and treated by employing the CPCM approach (ε(CH_3_CN) = 35.688) [44]. This computational model is very good at calculating hydricities, which was confirmed earlier [17,28,45]. The employed model is also benchmarked against the experimental hydricities of 21 known hydride donors taken from Ref. [45], providing the linear correlation function given in Equation (6) with R^2^ = 0.994 and the mean unsigned error of 1.8 kcal mol^−1^. This function was used for calculating the hydricities in this work.

ΔG*_H^−^_(exp) = 0.996 × ΔG*_HHR_ − 404.134 (6)

ΔG*_HHR_ = G*(DH) − G*(D^+^) (7)

The details of the calculations and the comparison with several other density functionals have been submitted to another journal.

The Gibbs energy was corrected for low-energy vibrations (<100 cm^−1^) according to the recommendations of Truhlar and coworkers [46]. The second-order perturbation energies were calculated using the NBO 3.1 routine within the Gaussian16 software, and the bond critical points were identified using AIM2000 software, version 2 [47,48,49]. The Multiwfn 3.8 program was used for the calculation of MCI indices from HF/6-31G(d) wavefunction and with iMCBOtype = 1 setting [50]. NICS(1)_zz_ values were taken from the NMR GIAO calculations at the B3LYP/6-311+G(d,p) level of theory at 1.0 Å above the geometrical center of each ring. Visualization of the geometries was done by Molden 6.9 and Avogadro^2^, version 1.102.1 [51,52,53].

## 5. Conclusions

The computational results presented here demonstrate two complementary strategies for enhancing CO_2_ reduction by benzimidazole-based hydride donors using superbasic groups.

Superbases act as strong electron-donating substituents that modulate hydricity, with their effectiveness being affected by steric and conformational factors. Changes in the aromaticity of the benzimidazole core, as interpreted by MCI aromaticity indices, provide a useful descriptor that correlates with hydricity, provided the size of the aromatic system remains essentially unchanged. Notably, α-substituted derivatives—especially bulky phosphazenes—show significant deviations from this correlation, highlighting steric limitations. These findings offer insight into the origin of hydricity and will support the rational design of improved donors.

The second approach involves lowering the barrier for CO_2_ reduction by incorporating a guanidinium cation as an auxiliary group that assists in CO_2_ binding through hydrogen bonding. This effect is particularly pronounced near the transition state, where an increase in the polarity of CO_2_ is expected due to its deformation. Our calculations show that the barrier can be lowered by more than 5 kcal mol^−1^ when a propyl linker is used.

Although these two approaches appear orthogonal, the applicability of hybrid systems (combining both a superbasic substituent and a guanidine auxiliary group) may be limited due to possible intramolecular proton transfer. Investigation of such systems, along with potential side processes, is currently underway.

In view of ongoing efforts to optimize the CO_2_ reduction process, our results expand the scope of potential hydride donors, particularly non-metallic ones. They also demonstrate that hydrogen bonding can facilitate the reduction process despite the nonpolarity of CO_2_.

## Data Availability

The original contributions presented in this study are included in the article/Supplementary Material. Further inquiries can be directed to the corresponding author.

## Supplementary Materials

The following supporting information can be downloaded at https://www.mdpi.com/article/10.3390/molecules31071167/s1. Table S1a. Electronic energies, Gibbs energies, Δ*G**_HHR_, and Δ*G**_H^−^_ calculated for hydride donors **1**–**11.** Table S1b. Electronic energies, Gibbs energies, Δ*G**_HHR_, and Δ*G**_H^−^_ calculated for hydride donors **12(a,b)H–22(a,b)H.** Table S2. Comparison of the C2-H bond lengths and Hirshfeld partial charges in the α- and β-substituted hydride donors belonging to the BIM and TAM groups. Figure S1. Correlation between experimental (in DMSO) and calculated (in CH_3_CN) hydricities (Δ*G*^*^_H^−^_) for the benzimidazole-based hydride donors **1H**, **2H,** and **4H**–**7H**. The hydricities were calculated according to the equation: Δ*G*^*^_H^−^_(calc) = 0.9969 × Δ*G*^*^_HHR_ − 405.4952. Figure S2. Graphical representation of HOMO calculated for the superbases **TMGH**, **P1H**, and **CPIH**. The electronic structure was calculated using CPCM(CH_3_CN)/ωB97xD/6-31+G(d,p) approach. Figure S3. Line plot of **16a^+^** calculated by the AIM2000 program. The bond critical points (BCP(3,−1)) located along the bond paths are marked by red dots. Figure S4. Correlation of NICS(1)_zz_ against MCI aromaticity indices for **12(a,b)H**–**16(a,b)H** (left) and **20(a,b)H**–**22(a,b)H** (right) and their cationic forms. Outlined circles and triangles stand for the β-substituted derivatives. Table S3. The NICS(1)_zz_ and MCI aromaticity indices calculated for hydride donors **1H**–**11H** and their cationic forms. Table S4. The NICS(1)_zz_ and MCI aromaticity indices calculated for hydride donors **12(a,b)H**–**22(a,b)H** and their cationic forms. Table S5a. Absolute and relative energies of 12 lowest-energy conformers of **23H_n1** obtained by GOAT(xTB) approach and their absolute and relative Gibbs energies calculated at CPCM(ACN)/wB97xD/aug-cc-pVTZ//CPCM(ACN)/wB97xD/6-31+G(d,p) level of theory. Table S5b. Absolute and relative energies of 12 lowest-energy conformers of **23H_n2** obtained by GOAT(xTB) approach and their absolute and relative Gibbs energies calculated at CPCM(ACN)/wB97xD/aug-cc-pVTZ//CPCM(ACN)/wB97xD/6-31+G(d,p) level of theory. Table S5c. Absolute and relative energies of 12 lowest-energy conformers of **23H_2_^+^** obtained by GOAT(xTB) approach and their absolute and relative Gibbs energies calculated at CPCM(ACN)/wB97xD/aug-cc-pVTZ//CPCM(ACN)/wB97xD/6-31+G(d,p) level of theory. Figure S5. Non-covalent interactions in three conformers of **23H_2_^+^** that were selected as the starting points in calculations of the reaction profiles for the reduction of CO_2_. Table S6. Energies (*E*_scf_, and *G*_tot_) and the hydricities (Δ*G**_H^−^_) calculated for **23H**, **23H_2_^+^**, **24H,** and **24H_2_^+^**. Table S7. Electronic energies, Gibbs energies, and Δ*G*_rel_ calculated for the selected stationary points along the reaction coordinate for the reduction of CO_2_.with **1H** and three conformers of **23H_2_^+^**. Table S8. Electronic energies, Gibbs energies, and Δ*G*_rel_ calculated for the selected stationary points along the reaction coordinate for the reduction of CO_2_, with three conformers of **24H_2_^+^**. Figure S6. Transition state structures identified along the reaction paths for the reduction of CO_2_ starting from three different conformers of **24H_2_^+^**. The red dotted lined shows the hydrogen bonding interactions and the hydride transfer coordinate. Table S9. Electronic energies (*E*_scf_), Gibbs energies (*G*_tot_), and Δ*G*_rel_ calculated for the selected stationary points along the reaction coordinate for the reduction of CO_2_ via carboxamidine intermediate starting from **23H**. The optimized geometries of all structures are collected in two files (Glasovac_hydricity.xyz and Glasovac_CO2_reduction.xyz), readable by Molden 6.9 and Mercury 3.10 software.

## References

[1] Crotwell A., Gatti L., Inness A., Koffi E., Labuschagne C., Lan X., Lee S., Luijkx I., Miller J., Agusti-Panareda A. (2025). The State of Greenhouse Gases in the Atmosphere Based on Global Observations Through 2024. WMO Greenh. Gas Bull..

[2] Anastas P.T., Warner J.C. (1998). Green Chemistry. Theory and Practice.

[3] GCCSI (2011). Accelerating the Uptake of CCS: Industrial Use of Captured Carbon Dioxide. https://perma.cc/KD4U-CRZM.

[4] Li L., Li X., Sun Y., Xie Y. (2022). Rational design of electrocatalytic carbon dioxide reduction for a zero-carbon network. Chem. Soc. Rev..

[5] Khalil M.T., Wu X., Ashraf S., Shen R., Liu S., Liu Y., Zhang H., Peng Z., Jiang J., Li B. (2025). Recent advancements in catalytic CO_2_ conversion to methanol: Strategies, innovations, and future directions. Green Chem..

[6] Choudhury J., Bhardwaj R., Kumar Mandal S. (2024). Hydride Transfer-Based CO_2_ Reduction Catalysis: Navigating Metal Hydride to Organic Hydride in the Catalytic Loop. Acc. Chem. Res..

[7] Kinoshita Y., Deromachi N., Kajiwara T., Koizumi T., Kitagawa S., Tamiaki H., Tanaka K. (2023). Photoinduced Catalytic Organic-Hydride Transfer to CO_2_ Mediated with Ruthenium Complexes as NAD^+^/NADH Redox Couple Models. ChemSusChem.

[8] Qi Y., Cheng W., Xu F., Chen S., Zhang S. (2018). Amino acids/superbases as eco-friendly catalyst system for the synthesis of cyclic carbonates under metal-free and halide-free conditions. Synth. Commun..

[9] Niemi T., Perea-Buceta J.E., Fernµndez I., Hiltunen O.-M., Salo V., Rautiainen S., Räisänen M.T., Repo T. (2016). A One-Pot Synthesis of *N*-Aryl-2-Oxazolidinones and Cyclic Urethanes by the Lewis Base Catalyzed Fixation of Carbon Dioxide into Anilines and Bromoalkanes. Chem. Eur. J..

[10] Aoyagi N., Furusho Y., Endo T. (2019). Efficient Catalysts of Acyclic Guanidinium Iodide for the Synthesis of Cyclic Carbonates from Carbon Dioxide and Epoxides Under Mild Conditions. Synthesis.

[11] Tamura M., Nakagawa Y., Tomishige K. (2022). Direct CO_2_ Transformation to Aliphatic Polycarbonates. Asian J. Org. Chem..

[12] Cui S., Borgemenke J., Liu Z., Li Y. (2019). Recent advances of “soft” bio-polycarbonate plastics from carbon dioxide and renewable bio-feedstocks via straightforward and innovative routes. J. CO_2_ Util..

[13] Pan Y.-Z., Lin X.-C., Liang Y., Xia Q., Cui F.-H., Tang H.-T., Pan Y.-M. (2025). Electrochemical Conversion of Low-Concentration CO_2_ Promoted by a Guanidine Polymer. JACS Au.

[14] Margetić D., Ishikawa T. (2009). Physico-Chemical Properties of Organosuperbases, in Superbases for Organic Synthesis.

[15] Das Neves Gomes C., Blondiaux E., Thuéry P., Cantat T. (2014). Metal-Free Reduction of CO_2_ with Hydroboranes: Two Efficient Pathways at Play for the Reduction of CO_2_ to Methanol. Chem. Eur. J..

[16] Nicholls R.L., McManus J.A., Rayner C.M., Morales-Serna J.A., White A.J.P., Nguyen B.N. (2018). Guanidine-Catalyzed Reductive Amination of Carbon Dioxide with Silanes: Switching Between Pathways and Suppressing Catalyst Deactivation. ACS Catal..

[17] Chen J., Yu H., Tan D., Lee R. (2023). Guanidine-Based Biomimetic Hydrides for Carbon Dioxide Reduction. Chem. Commun..

[18] Erhardt J.M., Grover E.R., Wuest J.D. (1980). Transfer of hydrogen from orthoamides. Synthesis, structure, and reactions of hexahydro-6bH-2a,4a,6a-triazacyclopenta[cd]pentalene and perhydro-3a,6a,9a-triazaphenalene. J. Am. Chem. Soc..

[19] Guo H., Liang Z., Guo K., Lei H., Wang Y., Zhang W., Cao R. (2022). Iron porphyrin with appended guanidyl group for significantly improved electrocatalytic carbon dioxide reduction activity and selectivity in aqueous solutions. Chin. J. Catal..

[20] Zhang Z., Yang Y., Wang J., Jing X., Duan C. (2025). Selective electrocatalytic reduction of carbon dioxide to methane using a guanidine-based metal–organic cage. Inorg. Chem. Front..

[21] Ilic S., Gesiorski J.L., Weerasooriya R.B., Glusac K.D. (2022). Biomimetic Metal-Free Hydride Donor Catalysts for CO_2_ Reduction. Acc. Chem. Res..

[22] DuBois D.L., Berning D.E. (2000). Hydricity of Transition-Metal Hydrides and Its Role in CO_2_ Reduction. Appl. Organomet. Chem..

[23] Miller A.J., Labinger J.A., Bercaw J.E. (2011). Trialkylborane-Assisted CO_2_ Reduction by Late Transition Metal Hydrides. Organometallics.

[24] Werra J.A., Wünsche M.A., Rathmann P., Mehlmann P., Löwe P., Dielmann F. (2020). Effective Control of the Electron-Donating Ability of Phosphines by Using Phosphazenyl and Phosphoniumylidyl Substituents. Z. Anorg. Allg. Chem..

[25] Weitkamp R.F., Neumann B., Stammler H.-G., Hoge B. (2021). Phosphorus-Containing Superbases: Recent Progress in the Chemistry of Electron-Abundant Phosphines and Phosphazenes. Chem. Eur. J..

[26] Weerasooriya R.B., Gesiorski J.L., Alherz A., Ilic S., Hargenrader G.N., Musgrave C.B., Glusac K.D. (2021). Kinetics of Hydride Transfer from Catalytic Metal-Free Hydride Donors to CO_2_. J. Phys. Chem. Lett..

[27] Barić D., Damjanović M., Glasovac Z., Despotović I., Kovačević B. (2026). Protonated bisphosphines: A new class of powerful hydride donors capable of CO_2_ reduction. Chem. Commun..

[28] Yeo C., Nguyen M., Wang L.-P. (2022). Benchmarking Density Functionals, Basis Sets, and Solvent Models in Predicting Thermodynamic Hydricities of Organic Hydrides. J. Phys. Chem. A.

[29] Briš A., Glasovac Z., Margetić D. (2021). Gas-phase basicity of cyclic guanidine derivatives—A DFT study. New J. Chem..

[30] Giambiagi M., de Giambiagi M.S., dos Santos Silva C.D., de Figueiredo A.P. (2000). Multicenter Bond Indices as a Measure of Aromaticity. Phys. Chem. Chem. Phys..

[31] Fallah-Bagher-Shaidaei H., Wannere C.S., Corminboeuf C., Puchta R., Schleyer P.v.R. (2008). Which NICS Aromaticity Index for Planar π Rings Is Best?. Org. Lett..

[32] Szatylowicz H., Jezuita A., Krygowski T.M. (2019). On the relations between aromaticity and substituent effect. Struct. Chem..

[33] Bannwarth C., Ehlert S., Grimme S. (2019). GFN2-xTB—An Accurate and Broadly Parametrized Self-Consistent Tight-Binding Quantum Chemical Method with Multipole Electrostatics and Density-Dependent Dispersion Contributions. J. Chem. Theory Comput..

[34] de Souza B. (2025). GOAT: A Global Optimization Algorithm for Molecules and Atomic Clusters. Angew. Chem. Int. Ed..

[35] Neese F., Wennmohs F., Becker U., Riplinger C. (2020). The ORCA quantum chemistry program package. J. Chem. Phys..

[36] Neese F. (2022). Software update: The ORCA program system—Version 5.0. WIREs Comput. Mol. Sci..

[37] Lim C.-H., Ilic S., Alherz A., Worrell B.T., Bacon S.S., Hynes J.T., Glusac K.D., Musgrave C.B. (2019). Benzimidazoles as Metal-Free and Renewable Hydrides for CO_2_ Reduction to Formate. J. Am. Chem. Soc..

[38] Harvey J.N., Himo F., Maseras F., Perrin L. (2019). Scope and Challenge of Computational Methods for Studying Mechanism and Reactivity in Homogeneous Catalysis. ACS Catal..

[39] Raczynska E.D., Gal J.-F., Maria P.-C. (2016). Enhanced Basicity of Push−Pull Nitrogen Bases in the Gas Phase. Chem. Rev..

[40] Kovačević B., Maksić Z.B. (2006). High basicity of phosphorus–proton affinity of tris-(tetramethylguanidinyl)phosphine and tris-(hexamethyltriaminophosphazenyl)phosphine by DFT calculations. Chem. Commun..

[41] Kaljurand I., Saame J., Rodima T., Koppel I., Koppel I.A., Kögel J.F., Sundermeyer J., Köhn U., Coles M.P., Leito I. (2016). Experimental Basicities of Phosphazene, Guanidinophosphazene, and Proton Sponge Superbases in the Gas Phase and Solution. J. Phys. Chem. A.

[42] Frisch M.J., Trucks G.W., Schlegel H.B., Scuseria G.E., Robb M.A., Cheeseman J.R., Scalmani G., Barone V., Petersson G.A., Nakatsuji H. (2019). Gaussian16. Rev C. 01.

[43] Chai J.-D., Head-Gordon M. (2008). Long-range corrected hybrid density functionals with damped atom-atom dispersion corrections. Phys. Chem. Chem. Phys..

[44] Marenich A.V., Cramer C.J., Truhlar D.G. (2009). Universal Solvation Model Based on Solute Electron Density and on a Continuum Model of the Solvent Defined by the Bulk Dielectric Constant and Atomic Surface Tensions. J. Phys. Chem. B.

[45] Ilic S., Alherz A., Musgrave C.B., Glusac K.D. (2018). Thermodynamic and kinetic hydricities of metal-free hydrides. Chem. Soc. Rev..

[46] Ribeiro R.F., Marenich A.V., Cramer C.J., Truhlar D.G. (2011). Use of Solution-Phase Vibrational Frequencies in Continuum Models for the Free Energy of Solvation. J. Phys. Chem. B.

[47] Reed A.E., Curtiss L.A., Weinhold F. (1988). Intermolecular interactions from a natural bond orbital, donor-acceptor viewpoint. Chem. Rev..

[48] Biegler-König F., Schönbohm J., Bayles D. (2001). AIM2000—A Program to Analyze and Visualize Atoms in Molecules. J. Comput. Chem..

[49] Biegler-König F., Schönbohm J. (2002). An Update to the AIM2000—Program for Atoms in Molecules. J. Comput. Chem..

[50] Lu T., Chen F. (2012). Multiwfn: A Multifunctional Wavefunction Analyzer. J. Comput. Chem..

[51] Schaftenaar G., Vlieg E., Vriend G. (2017). Molden 2.0: Quantum chemistry meets proteins. J. Comput.-Aided Mol. Des..

[52] Schaftenaar G., Noordik J.H. (2000). Molden: A pre- and post-processing program for molecular and electronic structures. J. Comput.-Aided Mol. Des..

[53] Hanwell M.D., Curtis D.E., Lonie D.C., Vandermeersch T., Zurek E., Hutchison G.R. (2012). Avogadro: An Advanced Semantic Chemical Editor, Visualization, and Analysis Platform. J. Chem. Inf..

