# Supplementary material for: DFT Investigation into the Role of Superbases as the Auxiliary Groups in CO2 Reduction
Source: Molecules. 2026 Apr 1;31(7):1167. doi: 10.3390/molecules31071167 (PMC13074656; doi:10.3390/molecules31071167)
Supplement: Supplementary file 1 [file molecules-31-01167-s001.zip › molecules-4203433_SupplMat.pdf]

Supplementary Material

for

DFT Investigation on the Role of Superbases as the Auxiliary Groups in CO_2 Reduction

Zoran Glasovac, Borislav Kovačević, and Davor Margetić

Table of contents:

S1. Energies of the benzimidazole-based hydride donors 2
S2. Superbases as substituents in benzimidazole-based hydride donors 6
S3. Aromaticity analysis....................7
S4. Conformations of **23H** and **$23H_2^+$** obtained by GOAT(xTB) calculations....................9
S5. Hidricity of the **23H, 24H** and their protonated forms (**$23H_2^+$** and **$24H_2^+$**) 16
S6. Energy data of the stationary points along the CO_2 reduction pathway 17
S7. CO_2 reduction pathway – the role of the guanidinium cation 18
S8. CO_2 reduction – Carbamate pathway 19
S9. Cartesian coordinates....................20

S1. Energies of the benzimidazole-based hydride donors

Table S1a. Electronic energies, Gibbs energies, ΔG^*_{HHR}, and $\mathbf{\Delta G^*_{H^-}}$ calculated for hydride donors **1 – 11.**[a]

Molecule	E_{scf}	G_{corr}	G_{tot}	ΔG^*_{HHR}	$\Delta G^*_{H^-}$(calc)[b]
**1H**	-499.00755	0.19064	-498.81691	448.7	42.7 (43.2)
**1+**	-498.28156	0.17973	-498.10183		
**2H**	-690.73923	0.23836	-690.50086	452.7	46.7 (49.1)
**2+**	-690.00966	0.23028	-689.77938		
**3H**	-805.27358	0.26788	-805.00570	452.0	45.1
**3+**	-804.54580	0.26036	-804.28544		
**4H**	-824.71712	0.30653	-824.41059	449.3	43.3 (45.8)
**4+**	-823.99291	0.29837	-823.69454		
**5H**	-1034.33484	0.32784	-1034.00700	444.8	38.7 (42.1)
**5+**	-1033.61844	0.32022	-1033.29822		
**6H**	-1688.72814	0.51085	-1688.21728	443.5	37.5 (40.5)
**6+**	-1688.01003	0.49955	-1687.51049		
**7H**	-1822.70585	0.57876	-1822.12709	438.2	32.2 (33.9)
**7+**	-1821.99604	0.56723	-1821.42881		
**8H**	-921.79027	0.31559	-921.47468	439.8	32.9
**8+**	-921.08164	0.30777	-920.77387		
**9H**	-1055.76674	0.38391	-1055.38284	434.4	27.6
**9+**	-1055.06631	0.37578	-1054.69052		
**10H**	-669.89216	0.25351	-669.63865	439.1	33.0
**10+**	-669.18299	0.24403	-668.93896		
**11H**	-594.87499	0.26161	-594.61338	429.7	22.8
**11+**	-594.18060	0.25194	-593.92866		

[a] E_{scf}, G_{corr}, and G_{tot} are given in a.u., while $\Delta_r G^*_{HHR}$ and $\Delta G^*_{H^-}$ are given in kcal mol^{-1}. [b] experimental hydricities measured in DMSO are given in parentheses.

Table S1b. Electronic energies, Gibbs energies, ΔG^*_{HHR}, and $\mathbf{\Delta G^*_{H^-}}$ calculated for hydride donors **12(a,b)H – 22(a,b)H.**[a]

Molecule	E_{scf}	G_{corr}	G_{tot}	ΔG^*_{HHR}	$\Delta G^*_{H^-}$(calc)
**12aH**	-632.97881	0.26017	-632.71864	448.7	42.6
**12a+**	-632.25411	0.25046	-632.00365		
**12bH**	-632.98053	0.25849	-632.72204	445.0	39.0
**12b+**	-632.25960	0.24679	-632.01281		
**13aH**	-860.42260	0.34613	-860.07648	445.8	39.8
**13a+**	-859.70381	0.33783	-859.36598		
**13bH**	-860.42294	0.34618	-860.07676	446.2	40.1
**13b+**	-859.70207	0.33634	-859.36573		
**14aH**	-859.22959	0.32746	-858.90213	445.9	39.8

**14a+**	-858.50927	0.31767	-858.19160		
**14bH**	-859.22938	0.32735	-858.90203	446.9	40.9
**14b+**	-858.50746	0.31764	-858.18982		
**15aH**	-1251.10703	0.56910	-1250.53793	444.8	38.7
**15a+**	-1250.38846	0.55932	-1249.82915		
**15bH**	-1251.10592	0.56791	-1250.53801	446.0	40.0
**15b+**	-1250.38608	0.55887	-1249.82720		
**16aH**	-1298.30404	0.41931	-1297.88474	442.6	36.5
**16a+**	-1297.58870	0.40923	-1297.17947		
**16bH**	-1298.30393	0.41895	-1297.88498	444.3	38.2
**16b+**	-1297.58584	0.40883	-1297.17701		
**17aH**	-1189.73400	0.45271	-1189.28130	436.6	29.7
**17a+**	-1189.03148	0.44588	-1188.58559		
**17bH**	-1189.74047	0.45224	-1189.28823	430.7	23.9
**17b+**	-1189.04466	0.44284	-1188.60182		
**18aH**	-1417.18281	0.54059	-1416.64222	436.6	29.8
**18a+**	-1416.47828	0.53184	-1415.94644		
**18bH**	-1417.18374	0.54009	-1416.64366	431.8	25.0
**18b+**	-1416.48762	0.53212	-1415.95550		
**19aH**	-1855.06166	0.61256	-1854.44910	433.4	26.6
**19a+**	-1854.36420	0.60581	-1853.75839		
**19bH**	-1855.06553	0.61298	-1854.45255	430.1	23.3
**19b+**	-1854.37225	0.60515	-1853.76710		
**20aH**	-803.86419	0.32306	-803.54113	438.8	32.0
**20a+**	-803.15469	0.31290	-802.84179		
**20bH**	-803.86523	0.32130	-803.54393	437.1	30.3
**20b+**	-803.15895	0.31163	-802.84731		
**21aH**	-1031.30877	0.40860	-1030.90017	436.7	29.8
**21a+**	-1030.60395	0.39967	-1030.20427		
**21bH**	-1031.30771	0.40910	-1030.89862	437.3	30.4
**21b+**	-1030.60163	0.39986	-1030.20177		
**22aH**	-1469.19027	0.48210	-1468.70817	434.3	27.5
**22a+**	-1468.48816	0.47212	-1468.01604		
**22bH**	-1469.18876	0.48155	-1468.70721	436.1	29.2
**22b+**	-1468.48449	0.47220	-1468.01229		

[a] E_{scf}, G_{corr}, **and** G_{tot} are given in a.u., while $\Delta_r G^*_{HHR}$ and $\Delta G^*_{H^-}$ are given in kcal mol^{-1}. [b] experimental hydricities measured in DMSO are given in parentheses.

Table S2. Comparison of the C2-H bond lengths and Hirshfeld partial charges in the α– and β–substituted hydride donors belonging to the BIM and TAM groups.[a]

Molecule	*d*(C2-H)	q(C2)	q(H)	Δq(C2)	*d*(C2-H)	q(C2)	q(H)	Δq(C2)
**BIM group**								
	**α– substituted derivatives**				**β – substituted derivatives**			
**1H**	1.115	0.071	0.019	0.099				
**1**$^{+}$		0.170						
**12(a,b)H**	1.114	0.070	0.019	0.096	1.116	0.069	0.017	0.092
**12(a,b)**$^{+}$	-	0.166				0.161		
**13(a,b)H**	1.116	0.067	0.016	0.084	1.116	0.069	0.017	0.094
**13(a,b)**$^{+}$		0.161				0.163		
**14(a,b)H**	1.116	0.068	0.016	0.095	1.116	0.069	0.017	0.094
**14(a,b)**$^{+}$		0.163				0.163		
**15(a,b)H**	1.116	0.071	0.015	0.087	1.116	0.068	0.016	0.093
**15(a,b)**$^{+}$		0.158				0.161		
**16(a,b)H**	1.117	0.065	0.013	0.092	1.117	0.067	0.015	0.091
**16(a,b)**$^{+}$		0.157				0.158		
**TAM group**								
**10H**	1.131	0.087	0.001	0.102				
**10**$^{+}$		0.189						
**20(a,b)H**	1.131	0.086	0.000	0.103	1.131	0.085	-0.001	0.100
**20(a,b)**$^{+}$		0.189				0.185		
**21(a,b)H**	1.132	0.084	-0.002	0.100	1.131	0.085	-0.001	0.100
**21(a,b)**$^{+}$		0.184				0.185		
**22(a,b)H**	1.133	0.082	-0.005	0.100	1.132	0.083	-0.003	0.100
**22(a,b)**$^{+}$		0.182				0.183		

a Bond lengths *d* are given in Ångstrøms and partial charges are given in |*e*|. Hirshfeld partial charges were calculated for CPCM(ACN)/wB97xD/6-31+G(d,p) wavefunction.

The calculated partial charges within either BIM or TAM group of structures vary only slightly and below the limit of the expected accuracy of the method. Therefore, no interpratation of the hydricity changes based on the partial charges were done.

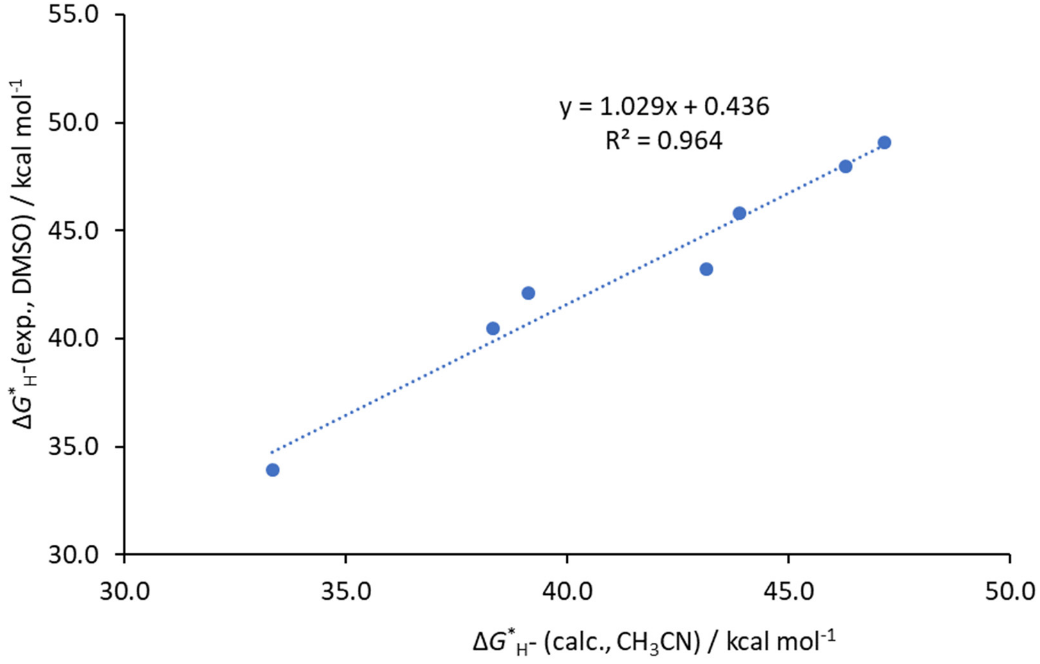

Figure S1. Correlation between experimental (in DMSO) and calculated (in CH_3CN) hydricities ($\Delta G^*_{H^-}$) for the benzimidazole-based hydride donors **1H**, **2H,** and **4H** – **7H**. The hydricities were calculated according to the equation: $\Delta G^*_{H^-}(calc) = 0.9969 \times \Delta G^*_{HHR} - 405.4952$.

The ΔG^*_{HHR} values were calculated as given in equation (S1).

$$\Delta G^*_{HHR}(XH) = G(X^+) - G(XH) \qquad (S1)$$

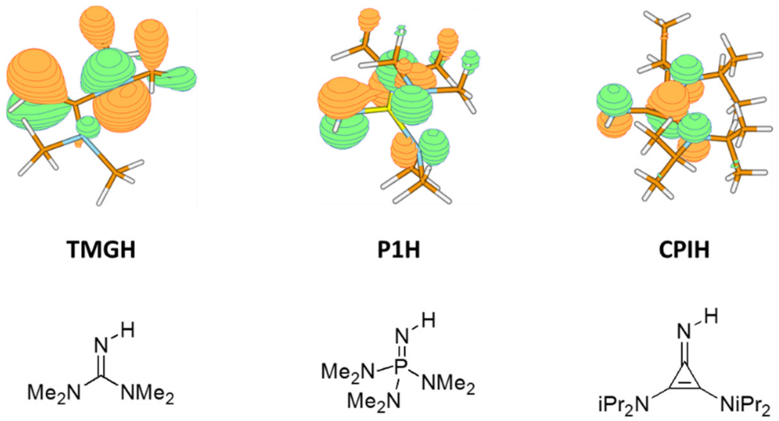

Figure S2. Graphical representation of HOMO calculated for the superbases **TMGH**, **P1H**, and **CPIH**. The electronic structure was calculated using CPCM(CH_3CN)/ωB97xD/6-31+G(d,p) approach.

S2. Superbases as substituents in benzimidazole-based hydride donors

Line plot of the critical points found in **11aP1**⁺ calculated by the AIM2000 program.

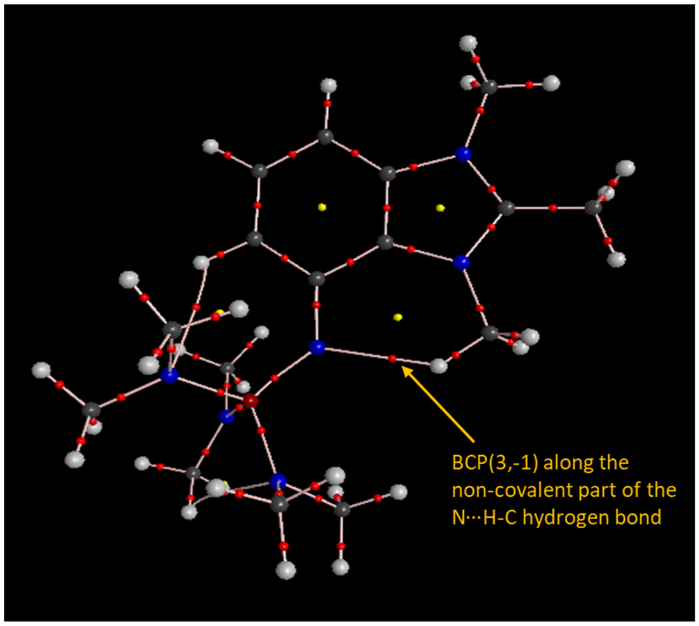

Figure S3. Line plot of **16a**⁺ calculated by the AIM2000 program. The bond critical points (BCP(3,-1)) located along the bond paths are marked by red dots.

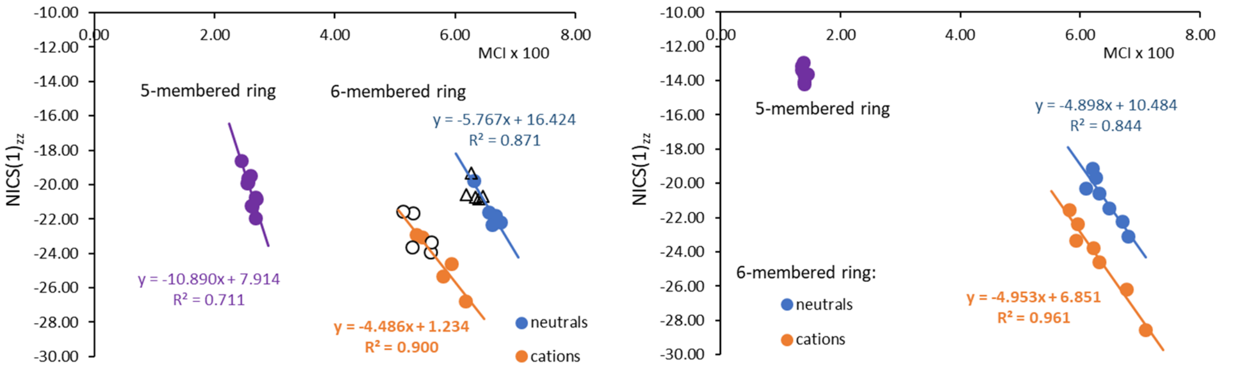

Figure S4. Correlation of NICS(1)zz against MCI aromaticity indices for **12(a,b)H – 16(a,b)H** (left) and **20(a,b)H–22(a,b)H** (right) and their cationic forms. Outlined circles and triangles stand for the β-substituted derivatives.

S3. Aromaticity analysis

Table S3. The NICS(1)zz and MCI aromaticity indices calculated for hydride donors **1H – 11H** and their cationic forms.[a]

Molecule	$NICS(1)_{zz}$ 6-memb.	$NICS(1)_{zz}$ 5-memb.	MCI 6-memb.	MCI 5-memb.	$\Delta(MCI)_6$ 6-memb.	$\Delta(MCI)_5$ 5-memb.
**1H**	-23.4	2.3	6.89	0.02	-0.30	2.59
**1⁺**	-30.0	-21.7	6.59	2.61		
**2H**	-23.6	1.7	6.87	0.07	-0.32	2.58
**2⁺**	-29.8	-21.5	6.55	2.65		
**3H**	-23.5	1.8	6.86	0.07	-0.28	2.51
**3⁺**	-29.4	-20.0	6.57	2.59		
**4H**	-23.4	1.8	6.83	0.07	-0.21	2.39
**4⁺**	-29.4	-20.0	6.62	2.46		
**5H**	-22.7	2.5	6.75	0.09	-0.19	2.58
**5⁺**	-29.4	-21.4	6.56	2.66		
**6H**	-21.5	1.3	7.06	0.04	-0.34	1.39
**6⁺**	-30.2	-21.9	6.72	1.43		
**7H**	-21.4	1.5	7.06	0.03	-0.19	1.15
**7⁺**	-29.1	-18.3	6.87	1.18		
**8H**	-20.6	1.6	6.94	0.05	-0.23	1.44
**8⁺**	-30.8	-21.5	6.71	1.49		
**9H**	-20.6	1.3	6.93	0.04	-0.09	1.20
**9⁺**	-29.7	-18.1	6.84	1.24		
**10H**	-23.1	1.7	6.80	0.02	0.28	1.37
**10⁺**	-28.6	-14.2	7.08	1.39		
**11H**	n/a	-1.2	n/a	0.02	n/a	2.84
**11⁺**	n/a	-16.5	n/a	2.86		

[a] $NICS(1)_{zz}$ and MCI aromaticity indices were calculated at the GIAO/B3LYP/6-311+G(d,p) and HF/6-31G(d) level of theory, respectively.

Table S4. The NICS(1)zz and MCI aromaticity indices calculated for hydride donors **12(a,b)H – 22(a,b)H** and their cationic forms.[a]

Molecule	$NICS(1)_{zz}$ 6-memb.	$NICS(1)_{zz}$ 5-memb.	MCI 6-memb.	MCI 5-memb.	$\Delta(MCI)_6$ 6-memb.	$\Delta(MCI)_5$ 5-memb.
**12aH**	-22.2	2.8	6.75	0.04	-0.57	2.64
**12a⁺**	-26.8	-21.9	6.18	2.68		
**12bH**	-20.6	2.2	6.18	0.02	-0.90	2.52
**12b⁺**	-23.6	-19.9	5.28	2.54		
**13aH**	-21.6	2.0	6.56	0.03	-0.77	2.59
**13a⁺**	-25.3	-21.3	5.79	2.62		
**13bH**	-20.8	1.9	6.39	0.01	-0.81	2.53
**13b⁺**	-23.9	-19.8	5.58	2.55		

**14aH**	-21.8	1.9	6.67	0.03	-0.73	2.59
**14a**$^{+}$	-24.6	-21.2	5.94	2.61		
**14bH**	-20.7	1.9	6.46	0.01	-0.86	2.54
**14b**$^{+}$	-23.3	-19.6	5.60	2.55		
**15aH**	-22.3	1.0	6.62	0.00	-1.26	2.68
**15a**$^{+}$	-22.9	-20.8	5.36	2.69		
**15bH**	-20.7	1.6	6.34	0.01	-1.17	2.56
**15b**$^{+}$	-21.6	-18.6	5.17	2.56		
**16aH**	-19.7	2.4	6.30	0.02	-0.86	2.66
**16a**$^{+}$	-23.1	-20.7	5.44	2.68		
**16bH**	-19.3	1.9	6.27	0.00	-1.14	2.58
**16b**$^{+}$	-21.5	-19.5	5.13	2.59		
**17aH**	-20.0	1.2	6.85	0.03	-0.53	1.17
**17a**$^{+}$	-25.2	-16.6	6.32	1.20		
**17bH**	-18.0	1.3	6.20	0.03	-0.59	1.16
**17b**$^{+}$	-24.2	-16.7	5.61	1.19		
**18aH**	-19.7	0.6	6.67	0.01	-0.58	1.15
**18a**$^{+}$	-26.7	-17.2	6.09	1.16		
**18bH**	-18.5	0.9	6.47	0.02	-0.56	1.18
**18b**$^{+}$	-24.8	-17.2	5.91	1.19		
**19aH**	-18.2	1.2	6.39	0.00	-0.76	1.17
**19a**$^{+}$	-22.0	-15.5	5.63	1.17		
**19bH**	-22.4	-16.8	6.30	0.02	-0.80	1.18
**19b**$^{+}$	-16.9	1.2	5.51	1.20		
**20aH**	-22.2	2.0	6.70	0.03	0.07	1.36
**20a**$^{+}$	-26.2	-13.7	6.77	1.39		
**20bH**	-20.3	1.7	6.09	0.01	-0.17	1.33
**20b**$^{+}$	-23.3	-13.1	5.93	1.35		
**21aH**	-21.5	1.5	6.48	0.02	-0.17	1.37
**21a**$^{+}$	-24.6	-14.0	6.31	1.40		
**21bH**	-20.6	1.6	6.31	0.01	-0.10	1.34
**21b**$^{+}$	-23.8	-13.4	6.22	1.35		
**22aH**	-19.6	1.9	6.25	0.02	-0.30	1.42
**22a**$^{+}$	-22.4	-13.6	5.95	1.44		
**22bH**	-19.1	1.6	6.20	0.00	-0.38	1.37
**22b**$^{+}$	-21.5	-12.9	5.82	1.37		

[a] $NICS(1)_{zz}$ and MCI aromaticity indices were calculated at the GIAO/B3LYP/6-311+G(d,p) and HF/6-31G(d) level of theory, respectively.

S4. Conformations of 23H and $23H_2^+$ obtained by GOAT(xTB) calculations

23H N1 conformer

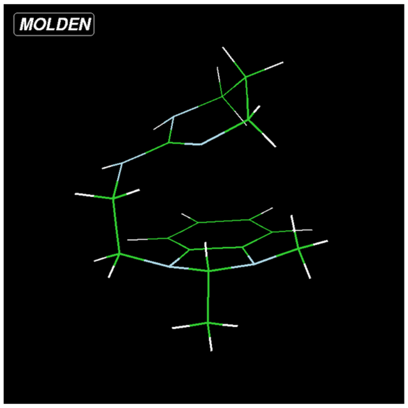

conf1

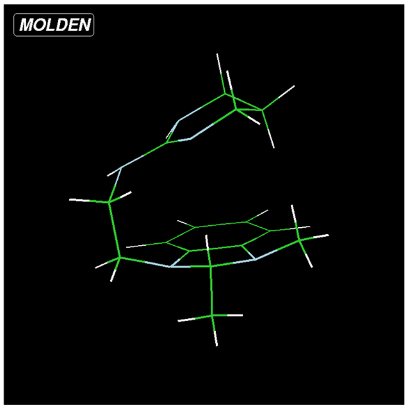

conf2

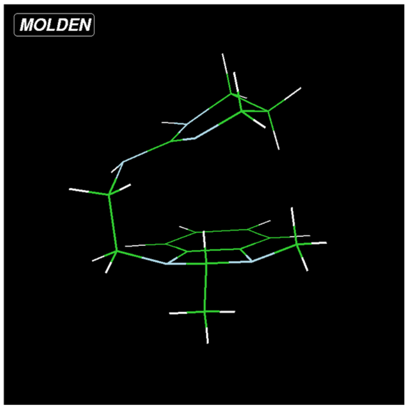

conf3

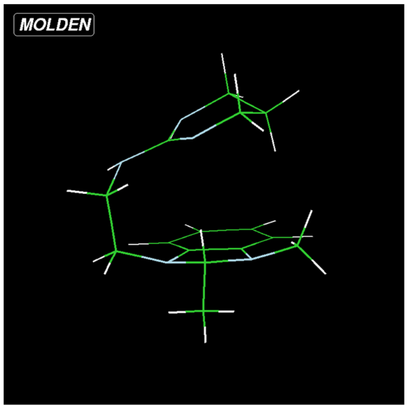

conf4

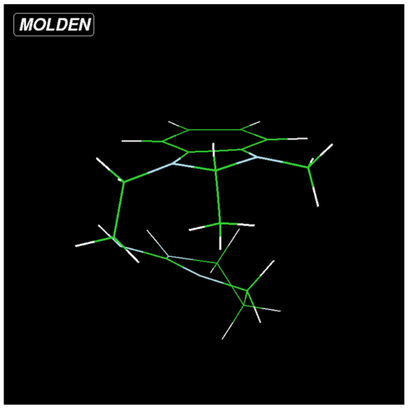

conf5

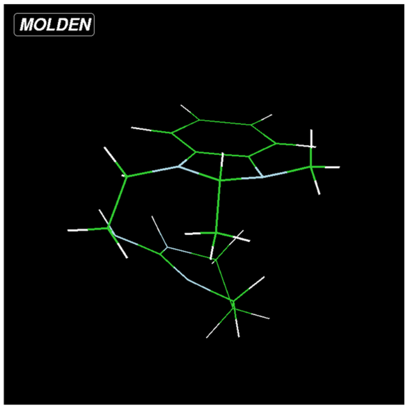

conf6

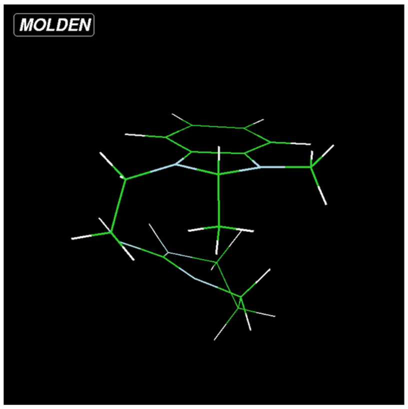

conf7

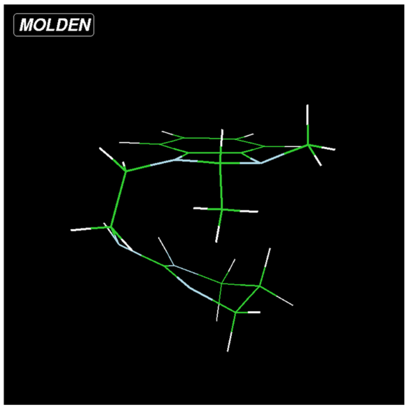

conf8

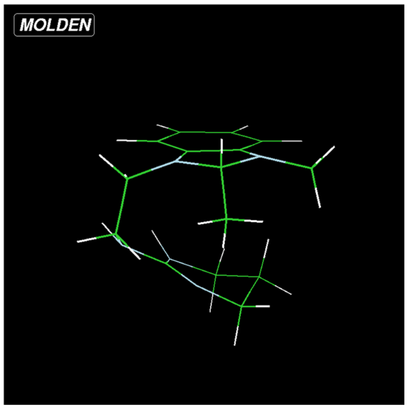

conf9

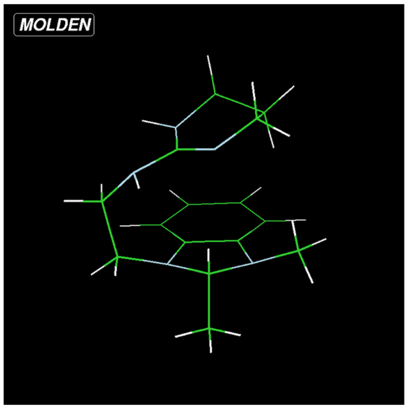

conf10

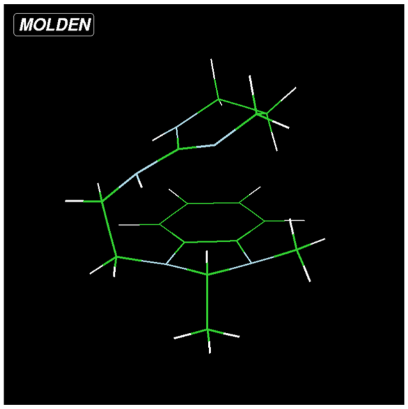

conf11

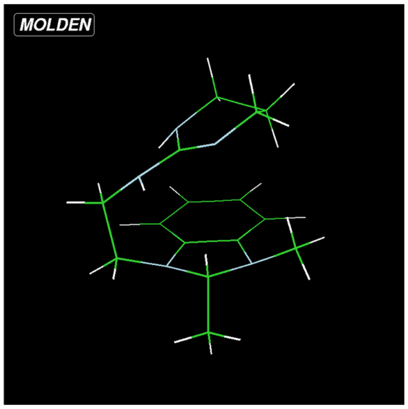

conf12

Table S5a. Absolute and relative energies of 12 lowest-energy conformers of **23H_n1** obtained by GOAT(xTB) approach and their absolute and relative Gibbs energies calculated at CPCM(ACN)/wB97xD/aug-cc-pVTZ//CPCM(ACN)/wB97xD/6-31+G(d,p) level of theory.[a]

Conformation	E(xTB/GOAT) / a.u.		*G*(wB97xD) / a.u.	
**conf1** [b]	-58.52998	0.00	**-858.91721**	**0.00**
conf2	-58.52954	0.28	-858.91693	0.18
conf3	-58.52909	0.56	-858.91676	0.28
conf4	-58.52839	1.00	-858.91697	0.15
conf5	-58.52720	1.74	-858.91006	4.48
conf6	-58.52708	1.82	-858.91401	2.01
conf7	-58.52677	2.02	-858.91353	2.31
**conf8** [b]	-58.52657	2.14	**-858.91484**	**1.48**
conf9	-58.52655	2.15	-858.91476	1.54
conf10	-58.52653	2.16	-858.91582	0.87
conf11	-58.52614	2.41	-858.91625	0.60
conf12	-58.52610	2.43	-858.91567	0.97

[a] Relative energies were calculated against the most stable conformer identified by the GOAT(xTB) approach. [b] Conformations marked by bolded letters were used for further structural variations.

23H N2 conformer

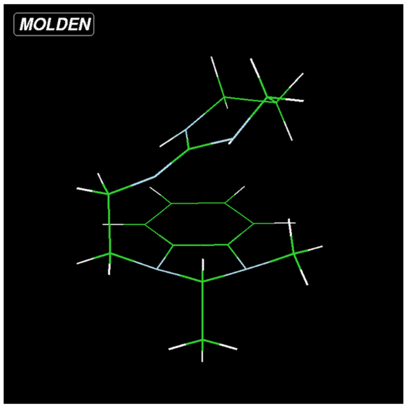

conf1

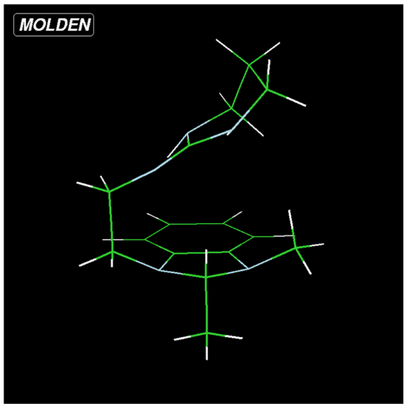

conf2

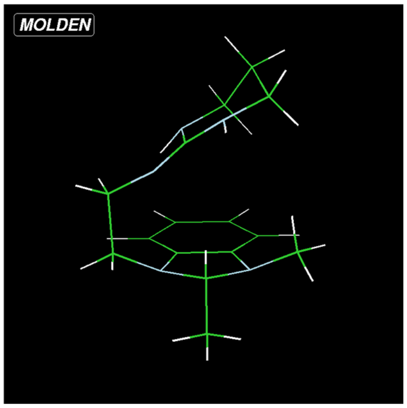

conf3

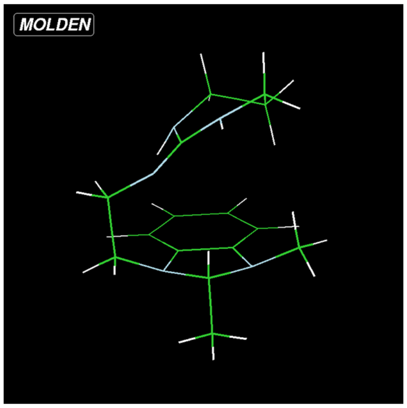

conf4

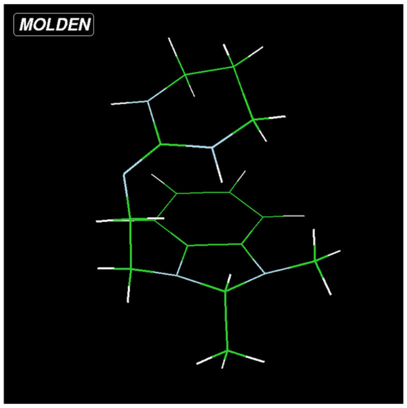

conf5

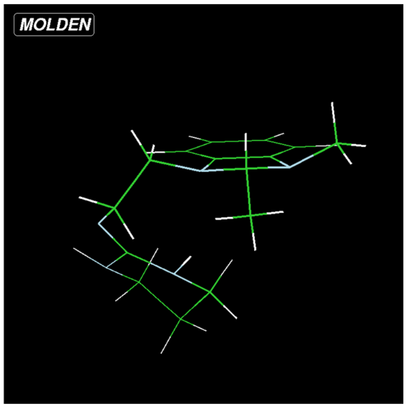

conf6

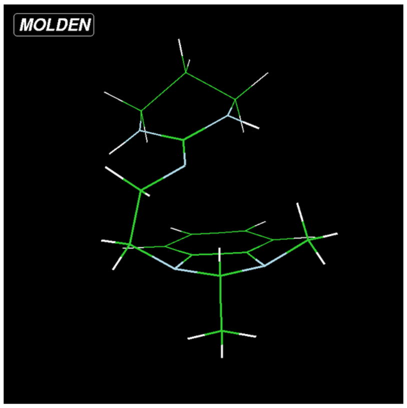

conf7

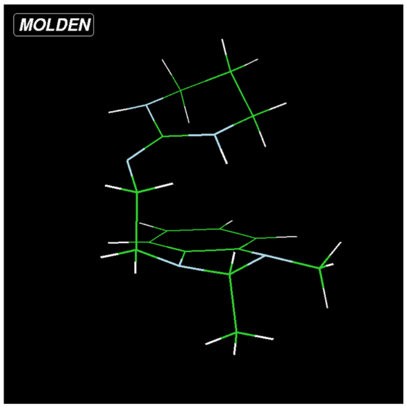

conf8

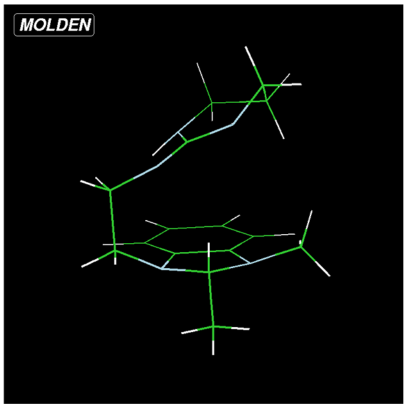

conf9

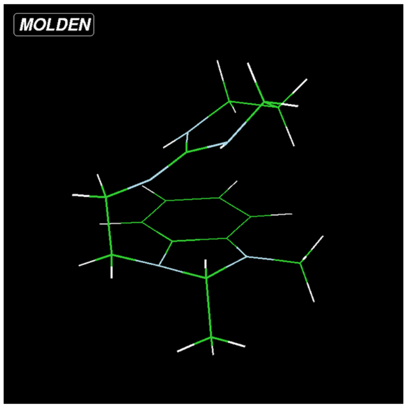

conf10

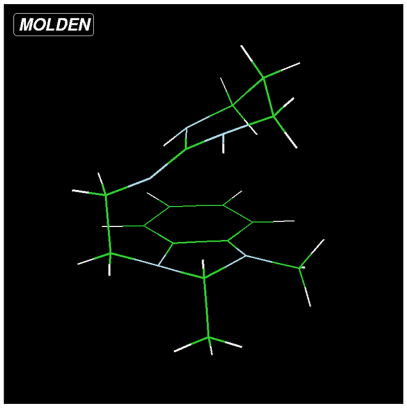

conf11

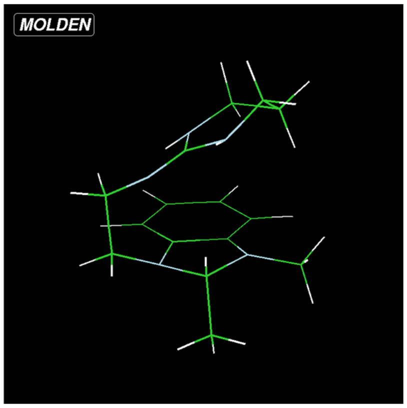

conf12

Table S5b. Absolute and relative energies of 12 lowest-energy conformers of **23H_n2** obtained by GOAT(xTB) approach and their absolute and relative Gibbs energies calculated at CPCM(ACN)/wB97xD/aug-cc-pVTZ//CPCM(ACN)/wB97xD/6-31+G(d,p) level of theory.[a]

Conformation	E(xTB/GOAT) / a.u.		*E*(wB97xD) / a.u.	
conf1	-58.51304	10.63	-858.91517	1.28
conf2	-58.51224	11.13	-858.91541	1.13
**conf3**[b]	-58.51177	11.43	**-858.91550**	**1.07**
conf4	-58.51167	11.49	-858.91501	1.38
conf5	-58.51133	11.70	-858.91396	2.04
**conf6**[b]	-58.51133	11.70	**-858.91578**	**0.89**
conf7	-58.51131	11.72	-858.91193	3.31
conf8	-58.51116	11.81	-858.91200	3.27
conf9	-58.51109	11.86	-858.91412	1.94
conf10	-58.51104	11.88	-858.91437	1.78
conf11	-58.51104	11.88	-858.91375	2.17
conf12	-58.51104	11.89	-858.91420	1.89

[a] Relative energies were calculated against the most stable conformer of **23H_n1** tautomer (Table S6a) identified by GOAT(xTB) approach. [b] Conformations marked by bolded letters were used for further structural variations.

$23H_2^+$

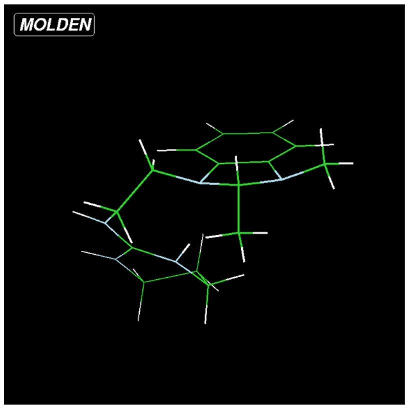

conf1

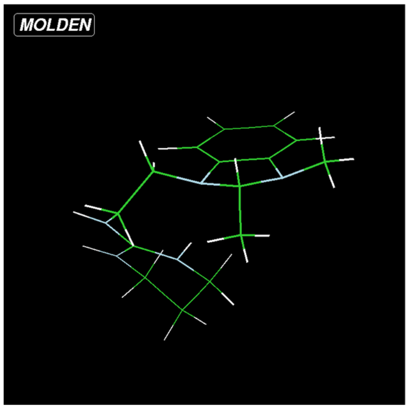

conf2

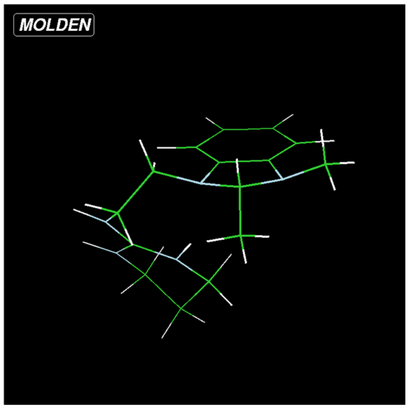

conf3

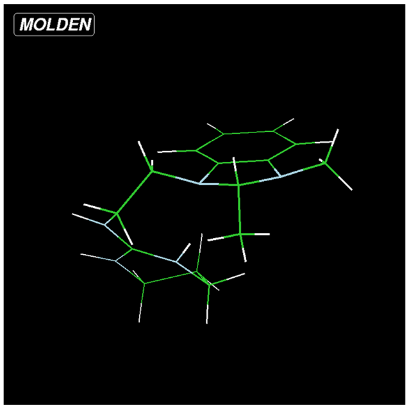

conf4

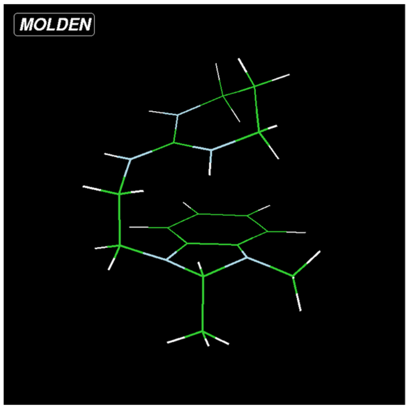

conf5

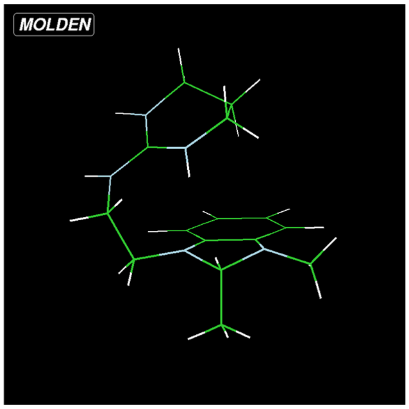

conf6

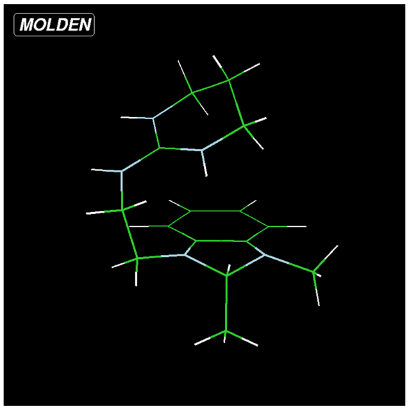

conf7

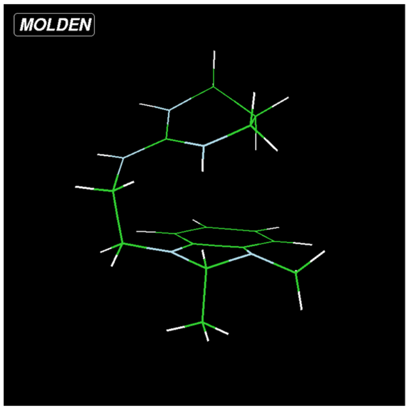

conf8

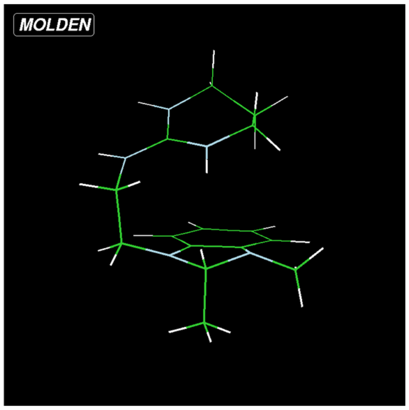

conf9

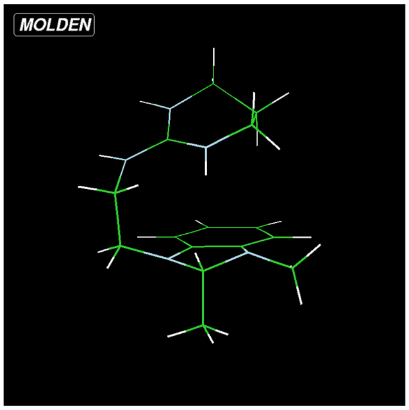

conf10

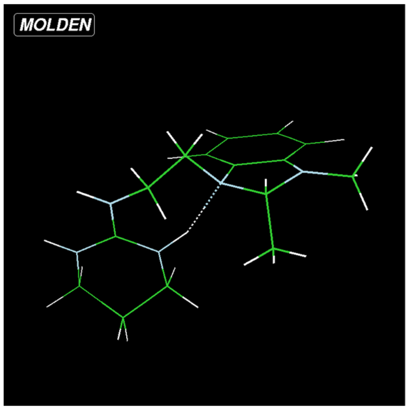

conf11

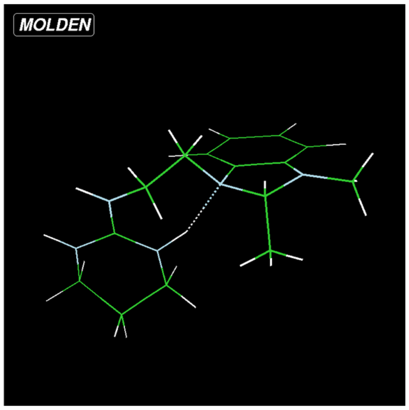

conf12

Table S5c. Absolute and relative energies of 12 lowest-energy conformers of **23H**$_2^{+}$ obtained by GOAT(xTB) approach and their absolute and relative Gibbs energies calculated at CPCM(ACN)/wB97xD/aug-cc-pVTZ//CPCM(ACN)/wB97xD/6-31+G(d,p) level of theory.[a]

Conformation	E(xTB/GOAT) / a.u.		*E*(wB97xD) / a.u.	
conf1	-58.66050	0.00	-859.38549	0.00
**conf2** [b]	-58.65967	0.51	-859.38627	**-0.49**
conf3	-58.65966	0.52	-859.38626	-0.48
conf4	-58.65943	0.67	-859.38541	0.05
conf5	-58.65866	1.15	-859.38098	2.83
**conf6** [b]	-58.65840	1.31	**-859.38188**	**2.27**
conf7	-58.65821	1.44	-859.38169	2.39
conf8	-58.65816	1.47	-859.38158	2.46
conf9	-58.65813	1.48	-859.38210	2.13
conf10	-58.65812	1.49	-859.38182	2.30
conf11	-58.65789	1.64	-859.38090	2.88
conf12	-58.65789	1.64	-859.37784	4.80

[a] Relative energies were calculated against the most stable conformer identified by GOAT(xTB) approach.
[b] Conformations marked by bolded letters were used for further structural variations.

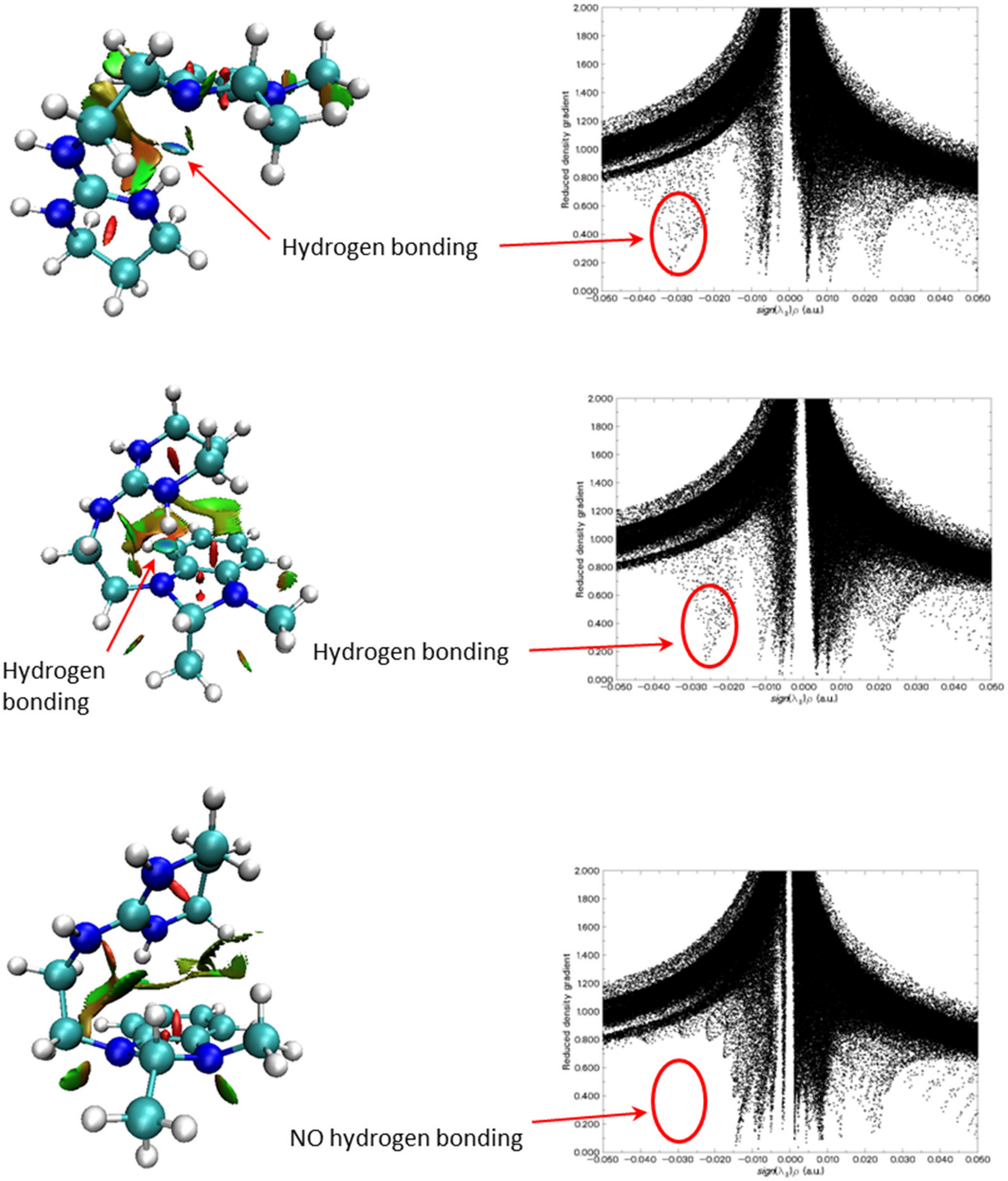

Figure S5. Non-covalent interactions in three conformers of **$23H_2^+$** that were selected as the starting points in calculations of the reaction profiles for the reduction of CO_2.

S5. Hidricity of the 23H, 24H and their protonated forms ($23H_2^+$ and $24H_2^+$)

Table S6. Energies (E_{scf}, and G_{tot}) and the hydricities ($\Delta G^*_{H^-}$) calculated for **23H**, **$23H_2^+$**, **24H,** and **$24H_2^+$** [a]

Structure	E_{scf}	G_{corr}	G_{tot}	ΔG_{HHR} (DH)	$\Delta G^*_{H^-}$(DH) [b]
**23H**	-859.25006	0.33277	-858.91729		
**23^+**	-858.52856	0.32386	-858.20471	447.2	41.1
**23^+_app[b]**	-858.52180	0.32228	-858.19952	450.4	44.3
**$23H_2^+$**	-859.73281	0.34640	-859.38641		
**$23H^{2+}$**	-858.99765	0.33916	-858.65848	456.8	50.7
**$23H^{2+}$_app[b]**	-858.99237	0.33593	-858.65644	458.1	52.0
**24H**	-898.56744	0.35998	-898.20745		
**24^+**	-897.84389	0.35333	-897.49055	449.9	43.8
**24^+_app[b]**	-897.83583	0.34551	-897.49032	450.0	43.9
**$24H_2^+$**	-899.04724	0.37198	-898.67526		
**$24H^{2+}$**	-898.31891	0.36573	-897.95318	453.1	47.0
**$24H^{2+}$_app[b]**	-898.31375	0.36272	-897.95103	454.5	48.4

[a] E_{scf}, G_{corr}, and G_{tot} are given in Hartrees, while ΔG_{HHR} and $\Delta G^*_{H^-}$ are given in kcal mol^{-1}; [b] $\Delta G^*_{H^-}$ values refer to the hydride form (**DH** = **23H**, **$23H_2^+$**, **24H,** or **$24H_2^+$**), and they were calculated using the equation: $\Delta G^*_{H^-} = 0.996 \times \Delta G_{HHR} - 404.13$ (see the main text); [c] the label "_app" stands for all-antiperiplanar conformation with no intramolecular hydrogen bonds.

S6. Energy data of the stationary points along the CO_2 reduction pathway

Table S7. Electronic energies, Gibbs energies, and ΔG_{rel} calculated for the selected stationary points along the reaction coordinate for the reduction of CO_2.with **1H** and three conformers of **$23H_2^+$**.[a]

Structure	E_{scf}	G_{corr}	G_{tot}	ΔG_{rel} [b]
**CO_2**	-188.60059	-0.00902	-188.60961	
**1H**	-499.00755	0.19064	-498.81691	-13.1
**1H_CO_2** (10Å)	-687.60807	0.20246	-687.40562	0.0
**1H_CO_2**	-687.61216	0.20796	-687.40420	0.9
**1H_TS1**	-687.57112	0.20111	-687.37001	22.3
**1H_FA**	-687.61263	0.20757	-687.40506	0.4
**c1 conformer**				
**$23H_2^+$(c1)**	-859.73281	0.35806	-859.37475	-13.6
**$23H_2^+$_CO_2 (c1)** (10Å)	-1048.33420	0.37158	-1047.96262	0.0
**$23H_2^+$_CO_2(c1)**	-1048.33806	0.37412	-1047.96394	-0.8
**$23H_2^+$_TS1(c1)**	-1048.29325	0.36922	-1047.92403	24.2
**$23H_2^+$_FA(c1)**	-1048.33172	0.37444	-1047.95728	3.4
**c2 conformer**				
**$23H_2^+$(c2)**	-859.73079	0.35741	-859.37338	-12.8
**$23H_2^+$_CO_2(c2)**	-1048.33591	0.37480	-1047.96111	0.9
**$23H_2^+$_TS1(c2)**	-1048.29761	0.36635	-1047.93126	19.7
**$23H_2^+$_FA(c2)**	-1048.34909	0.37392	-1047.97517	-7.9
**c3 conformer**				
**$23H_2^+$(c3)**	-859.73080	0.35815	-859.37265	-12.3
**$23H_2^+$_CO_2(c3)**	-1048.33252	0.37447	-1047.95805	2.9
**$23H_2^+$_TS1(c3)**	-1048.29548	0.36777	-1047.92771	21.9
**$23H_2^+$_FA(c3)**	-1048.34884	0.37336	-1047.97548	-8.1
**$23H_2^+$_FA_{2HB}(c3)**[c]	-1048.35636	0.37266	-1047.98371	-13.2

[a] E_{scf}, G_{corr}, and G_{tot} are given in Hartrees, while ΔG_{rel} is given in kcal mol^{-1}. G_{corr} values are corrected for low-energy vibrations as described in the Materials and methods section; [b] **1H_CO_2** (10Å) and **$23H_2^+$_CO_2 (c1)** (10Å) were taken as the reference structures; [c] FA_{2HB} label indicates structure with optimal hydrogen bonding between the guanidinium subunit and the formate anion (FA).

S7. CO_2 reduction pathway – the role of the guanidinium cation

Table S8. Electronic energies, Gibbs energies, and ΔG_{rel} calculated for the selected stationary points along the reaction coordinate for the reduction of CO_2, with three conformers of **$24H_2^+$**.[a]

Structure	E_{scf}	G_{corr}	G_{tot}	ΔG_{rel} [b]
**c1 conformer**				
**$24H_2^+$(c1)**	-899.05027	0.38678	-898.66349	-14.4
**$24H_2^+$_CO_2(c1) (10Å)**	-1087.65095	0.40073	-1087.25022	0.0
**$24H_2^+$_CO_2(c1)**	-1087.65570	0.40176	-1087.25394	-2.3
**$24H_2^+$_TS1(c1)**	-1087.61195	0.39620	-1087.21575	21.6
**$24H_2^+$_FA(c1)**	-1087.65131	0.40074	-1087.25057	-0.2
c2 conformer				
**$24H_2^+$(c2)**	-899.05064	0.38705	-898.66359	-14.4
**$24H_2^+$_CO_2(c2)**	-1087.65271	0.401988	-1087.25072	-0.3
**$24H_2^+$_TS1(c2)**	-1087.61585	0.395143	-1087.22070	18.5
**$24H_2^+$_FA(c2)**	-1087.66781	0.399687	-1087.26813	-11.2
c3 conformer				
**$24H_2^+$(c3)**	-899.04843	0.385664	-898.66277	-13.9
**$24H_2^+$_CO_2(c3)**	-1087.65264	0.40165	-1087.25099	-0.5
**$24H_2^+$_TS1(c3)**	-1087.61809	0.39461	-1087.22348	16.8
**$24H_2^+$_FA(c3)** [c]	-1087.67345	0.40167	-1087.27178	-13.5

[a] E_{scf}, G_{corr}, and G_{tot} are given in Hartrees, while ΔG_{rel} is given in kcal mol^{-1}; G_{corr} values are corrected for low-energy vibrations as described in the Materials and methods section; [b] **$24H_2^+$_CO_2 (c1)** (10Å) was taken as the reference structures; [c] **$24H_2^+$_FA** structure achieved the optimal hydrogen bonding motif (two HBs) during the IRC calculations.

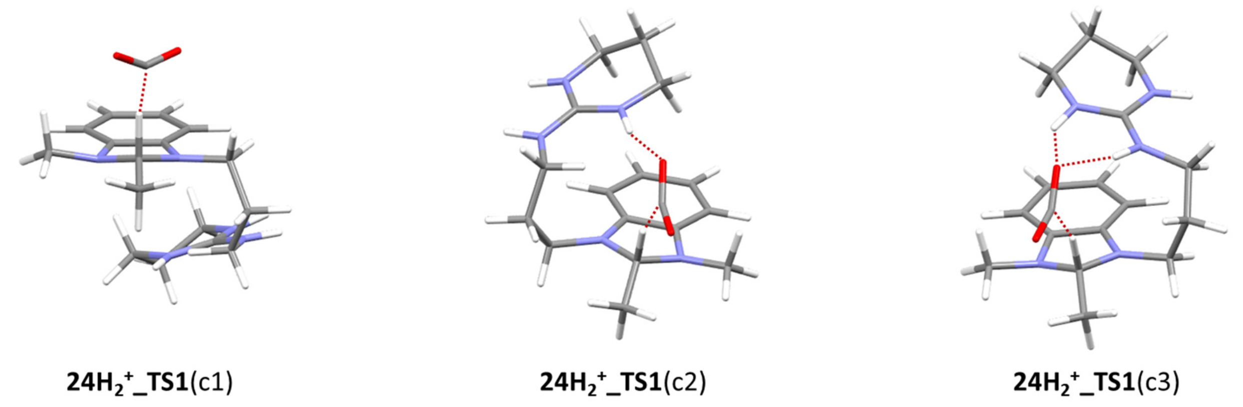

Figure S6. Transition state structures identified along the reaction paths for the reduction of CO_2 starting from three different conformers of **$24H_2^+$**. The red dotted lined shows the hydrogen bonding interactions and the hydride transfer coordinate.

S8. CO_2 reduction – Carbamate pathway

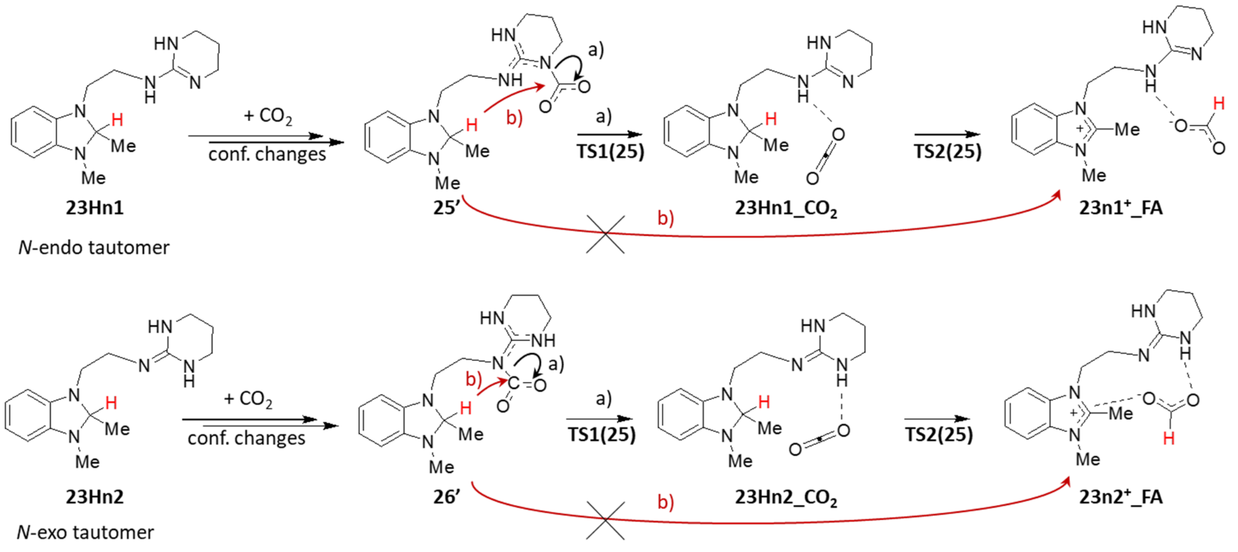

Table S9. Electronic energies (E_{scf}), Gibbs energies (G_{tot}), and ΔG_{rel} calculated for the selected stationary points along the reaction coordinate for the reduction of CO_2 via carboxamidine intermediate starting from **23H**.[a]

Structure	E_{scf}	G_{corr}	G_{tot}	ΔG_{rel} [b]
**N1 tautomer**				
**23Hn1**	-859.24745	0.34426	-858.90319	1.2
**25**	-1047.87597	0.36126	-1047.51472	0.0
**25'**	-1047.87121	0.36093	-1047.51028	2.8
**TS1(25)**	-1047.84896	0.35867	-1047.49029	15.3
**23Hn1_CO₂**	-1047.85248	0.35888	-1047.49359	13.3
**TS2(25)**	-1047.81202	0.35309	-1047.45893	35.0
**23n1⁺_FA**	-1047.86056	0.36048	-1047.50008	9.2
**N2-tautomer**				
**23Hn2**	-859.24614	0.34436	-858.90178	2.1
**26**	-1047.87437	0.35977	-1047.51460	0.1
**26'**	-1047.86943	0.36057	-1047.50886	3.7
**TS1(26)**	-1047.84729	0.35817	-1047.48912	16.1
**23Hn1_CO₂**	-1047.85115	0.35881	-1047.49235	14.0
**TS2(26)**	-1047.81122	0.35314	-1047.45807	35.5
**23n2⁺_FA**	-1047.85959	0.35991	-1047.49968	9.4

[a] E_{scf}, G_{corr}, and G_{tot} are given in Hartrees, while ΔG_{rel} is given in kcal mol^{-1}. G_{corr} values are corrected for low-energy vibrations as described in the Materials and methods section; [b] G_{tot} value of carboxamidine **25** was used as the reference structure; [c] FA_{2HB} label indicates structure with optimal hydrogen bonding between the guanidinium subunit and the formate anion (FA).

S9. Cartesian coordinates

Cartesian coordinates of all structures relevant for this paper are given in the form of two text files readable with the Molden or Mercury programs.

Glasovac_hydricity.xyz

Glasovac_CO2_reduction.xyz
